# Large-area patterning of full-color quantum dot arrays beyond 1000 pixels per inch by selective electrophoretic deposition

**DOI:** 10.1038/s41467-021-24931-x

**Published:** 2021-07-29

**Authors:** Jinyang Zhao, Lixuan Chen, Dongze Li, Zhiqing Shi, Pai Liu, Zhenlei Yao, Hongcheng Yang, Taoyu Zou, Bin Zhao, Xin Zhang, Hang Zhou, Yixing Yang, Weiran Cao, Xiaolin Yan, Shengdong Zhang, Xiao Wei Sun

**Affiliations:** 1 Key Laboratory of Energy Conversion and Storage Technologies, Ministry of Education, and Department of Electrical and Electronic Engineering, Southern University of Science and Technology, Shenzhen, Guangdong China; 2 School of Electronic and Computer Engineering, Peking University, Shenzhen, Guangdong China; 3 Shenzhen China Star Optoelectronics Semiconductor Display Technology Co., Ltd, Shenzhen, Guangdong China; 4 TCL Research, 1001 Zhongshan Park Road, Nanshan District, Shenzhen, China; 5 Shenzhen Planck Innovation Technologies Co. Ltd, Shenzhen, Guangdong China

**Keywords:** Electronic devices, Design, synthesis and processing, Quantum dots

## Abstract

Colloidal quantum dot (QD) emitters show great promise in the development of next-generation displays. Although various solution-processed techniques have been developed for nanomaterials, high-resolution and uniform patterning technology amicable to manufacturing is still missing. Here, we present large-area, high-resolution, full-color QD patterning utilizing a selective electrophoretic deposition (SEPD) technique. This technique utilizes photolithography combined with SEPD to achieve uniform and fast fabrication, low-cost QD patterning in large-area beyond 1,000 pixels-per-inch. The QD patterns only deposited on selective electrodes with precisely controlled thickness in a large range, which could cater for various optoelectronic devices. The adjustable surface morphology, packing density and refractive index of QD films enable higher efficiency compared to conventional solution-processed methods. We further demonstrate the versatility of our approach to integrate various QDs into large-area arrays of full-color emitting pixels and QLEDs with good performance. The results suggest a manufacture-viable technology for commercialization of QD-based displays.

## Abstract (web summary)

Colloidal quantum dots are promising for next-generation displays, yet the technology to realise high-resolution and uniform patterning is still scarce. Here, the authors report full-colour QD large area patterning by combining photolithography and selective electrophoretic deposition technique.

## Introduction

Semiconductor quantum dots (QDs) are promising optoelectronic materials because of their exceptional optoelectronic properties, such as size-controlled tunable emission wavelength, narrow emission spectrum, high luminescent efficiency, and compatibility with solution processing^1–3^. Accordingly, QDs have enormous potentials as an essential component in high-efficiency luminescent devices, light-emitting diodes, photovoltaic cells, photodetectors, and other related devices^4–6^. To integrate them with the above-mentioned solid-state devices, the QDs must be precisely and accurately patterned in predetermined locations^7–9^. So far, a variety of techniques have been applied to pattern functional nanomaterials, such as mask-based photolithography, inkjet printing, transfer printing, microcontact printing, and nanoimprint lithography, etc^10–22^. However, most of these methods suffer from drawbacks such as performance degradation by ultraviolet light and solvents, complex processing technology, long processing time, low efficiency, and poor repeatability. Therefore, improving and developing novel QD patterning technology is critical for commercially viable applications.

Colloidal QDs are generally composed of an inorganic semiconductor core and an organic ligand shell^23,24^. QDs are not stoichiometric in composition and their ligands can ionize in the solution, therefore they will be rich in either anion or cation species, i.e., QDs are consequently rich in either cations or anions on their surfaces^25–27^. Therefore, the presence of surface charges in QDs provides an effective way of electrically driving the movement of QDs in a solution and depositing them onto the surfaces of the electrodes of opposite polarity^28–30^. This approach of electrophoretic deposition (EPD) has shown great potential for the fabrication of large-area films as the photoanodes of quantum dot sensitized solar cells^31–34^. However, the technique still remains to be improved to resolve the problems such as low deposition selectivity, QDs adhering to both cathode and anode with non-uniform thickness, and hence the difficulty in multi-material integration. Thus, developing a new method of EPD that provides fast and precise deposition of large-area multicolor QD emissive patterns would be necessary, which would greatly facilitate the practical applications of QDs for displays, imaging, and photodetectors.

Here, we present a universal QD patterning technique using photolithography combined with mild and low-cost selective electrophoretic deposition (SEPD) to achieve large-area full-color QD patterning. This novel EPD process utilizes a high-accuracy microelectrode by photolithography to rapidly (a few seconds) fabricate QD patterns only on selective polarity electrodes. The deposited QD films with controlled, uniform feature sizes down to 2 µm and precisely modulated thickness can be patterned into pixel array with arbitrary yet predefined shapes beyond 1,000 pixels per inch (PPI). The surface morphology, packing density, and refractive index of deposited QD films are tunable by changing the electric field, enabling us to tailor the performance of QD devices and achieve higher luminous efficiency than conventional solution-processed methods. Based on these, we demonstrate the versatility of our approach to integrate QDs with different emission properties into large-area arrays to form full-color photoluminescence (PL) pixels, and further construct high-performance QD light-emitting diodes (QLEDs). Such devices developed with this approach are promising for next-generation displays with excellent optoelectronic performance and low-cost fabrication.

## Results and discussion

### Patterning of QDs array via SEPD

**Fig. 1:**
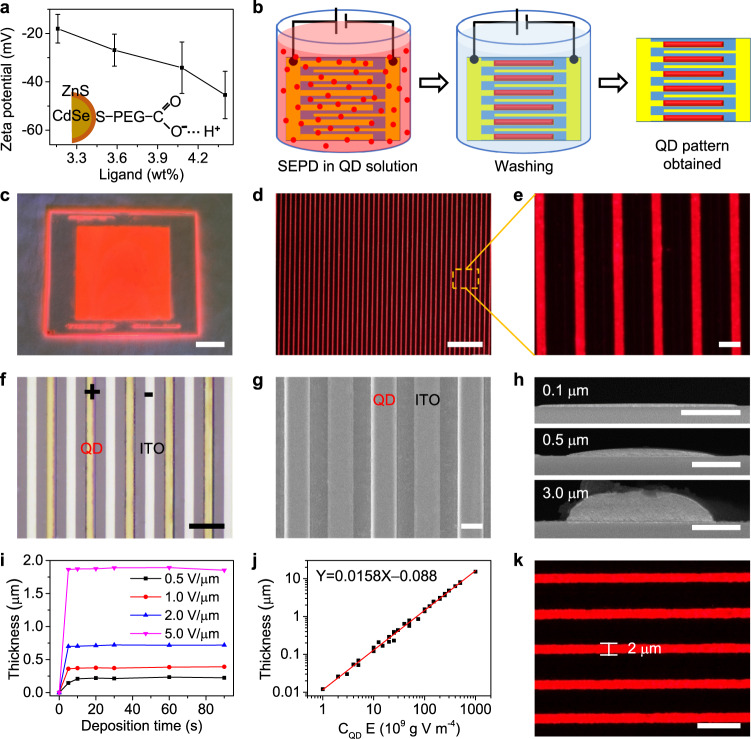
Patterning of QDs array via SEPD. **a** Zeta potentials of QDs capped with different ligand contents. The error bars are the standard deviation in measured zeta potentials for ten runs. Inset: schematic representation of a QD with ionized surface ligands. **b** Schematic illustration of the QDs patterning process on the in-plane parallel electrodes substrate. **c** Image of large-area ordered QD stripe pattern. Scale bar, 2 cm. **d**, **e** Microscopy images of the deposited QD stripe array showing the uniform size and a well-defined pattern. Scale bars are 200 and 20 μm, respectively. **f**, **g** Bright-field image and SEM image of QD pattern via SEPD. Scale bars are 50 and 10 μm, respectively. **h** Cross-sectional SEM images of QD stripes with different thickness. Scale bars, 5 μm. **i** Relationship between the deposited thickness of the QD stripe and deposition time at different electric fields. **j** Deposited QD stripe layer thickness as a function of electric field intensity (*E*) and QDs concentrations (*C*_QD_) product. **k** Fluorescence image of QD stripe array with a linewidth of ≈ 2 µm. Scale bar, 10 μm.

As well known, the surfaces of QDs can be charged by the ionization of the ligands (such as carboxylic-acid-, phosphonic-acid-, and thiol-terminated ligands) in polar solution^35^, which facilitates the electric field deposition of the QDs^25,36^. Therefore, colloidal CdSe/ZnS core/shell QD capped with polyethylene glycol (PEG)-COOH in propylene glycol methyl ether acetate (PGMEA) was selected because of its high PL quantum yield (PLQY) and solution-processability. To analyze and improve the surface charges, four QDs modified by PEG-COOH ligands with different contents were designed and synthesized. And their charge characteristics were estimated by measuring their zeta potentials and electrophoretic mobilities (Supplementary Table 1). As shown in Fig. 1a, the zeta potentials of all QDs are negative, indicating that these QDs are negatively charged by the ionized carboxylic acid due to the ligand ionization in PGMEA solution (Fig. 1a, inset). Moreover, the absolute value of zeta potential is increased with the increase of ligand content. This testifies that the charges on QDs mainly originate from ligand ionization and can be tuned by modifying the ligand. However, ligand modification influences PLQY of QD slightly, in our experiment, the QD with the highest PLQY was selected as the model compound for the construction of multicolor patterned structures.

Figure 1b schematically illustrates the process of patterning QDs on an in-plane interdigitated electrodes by SEPD. The electrode substrate was placed into the QDs solution. A DC electric field in the range of 0.5−10 V μm^−1^ was then applied for QD deposition. The electric field force drives the movement and deposition of QDs in solution onto the surfaces of the electrodes of opposite polarity. Then, patterning of QDs can be achieved by the SEPD of the charged QDs into a film of arbitrary size, shape following the electrode geometry. After a fixed time, the substrate was lifted out of the QD solution. With the voltage bias remaining on, the electrodes were washed by dipping into a pure solution for 10 s. This washing step removes aggregated QDs that were not well-bounded to the surface and QD-rich droplets. After SEPD, the substrate was dried in an oven at 80 °C for 20 min, and finally, the patterned QD film was obtained.

The as-fabricated QD stripe film can be patterned over a very large area, which is only limited by substrate size. Figure 1c shows the photograph of a 10 × 10 cm QD pattern deposited on a glass substrate with interdigitated ITO electrodes. Under ultraviolet (UV) irradiation, the QD patterns emit bright and uniform red fluorescence (Fig. 1d and Supplementary Fig. 1). The QD patterns are in a uniform geometric shape with precise positioning (Fig. 1e). As the bright field optical microscopy (OM) and scanning electron microscopy (SEM) images shown (Fig. 1f and g), QDs are only deposited on positive electrodes, which is in contrast with nothing on the negative electrodes. This result further indicates the surfaces of QDs are only negatively charged. A cross-sectional SEM and 3D OM images of deposited QD stripe pattern with different thickness are as shown in Fig. 1h and Supplementary Fig. 2, respectively. It can be observed that these deposited QD microstructures have close-packed arc cross-section structures without visible pinhole or surface defects, and the planeness of QDs patterns can be tuned to modify the interface and uniformity for optoelectronic devices by changing their thickness and linewidth (Supplementary Fig. 3).

The thickness of the SEPD QD microstructures can be controlled by varying the SEPD parameters, such as the electric field intensity, QD concentration, deposition time and so forth^28,32^. Figure 1i demonstrates the kinetic aspects of SEPD through deposited thickness against time. At a lower electric field (for example 0.5 V μm^−1^), the deposition rate is relatively slower. At a higher electric field, SEPD can be completed within 5 s due to the fast deposition rate of our method. Therefore, we set the deposition time to tens of seconds in our experiments, which is enough to complete the SEPD. Figure 1j demonstrates the thickness of QD patterns as a function of field intensity and various QD concentration products (Supplementary Fig. 4). With enough QDs in solution and for fixed deposition time, the film thickness monotonically increases with the electric field and QD concentration. The curve has a linear slope of about 1.58 × 10^−17^ m^5^ g^−1^ V^−1^. Therefore, the thickness of QD patterns can be precisely controlled in a large range from several nanometers to tens of microns through the combination of electric field and QD concentration, which not only can meet the thickness requirement for color conversion layer in LCDs, blue OLEDs, and micro-LEDs, but also the accuracy demand of QLED. The linewidth and space of the QD pattern mainly depend on the designed electrode. As shown in Supplementary Fig. 5, it demonstrates the QD microscale line array with different widths and gaps. We have further reduced the width of the ITO electrode to 2 μm by optimizing the process of photolithography (Supplementary Fig. 6), and the resulting QD stripe width is within 5% variation of the designed 2 μm width (Fig. 1k). Generally, the resolution limitation of the SEPD method depends on the sizes of patterned electrodes, which are limited by the processing method.

### Characteristic control of QD patterns

To directly observe the movement and deposition of QDs in solution under electric field, a glass cell with in-plane interdigital electrode substrate was used as a container to hold the QD solution and was placed under a micro-PL system (Supplementary Fig. 7a). Because of the high surface negative charges, the QDs can be driven by the electric field to move rapidly to the positive electrodes. As the Supplementary Movie 1 shown, within one second, all QDs have been depleted in the solution with an electric field of 5 V μm^−1^, which indicates the extremely fast processing speed of our SEPD method and the potential for fast electrophoretic displays. In addition, the QDs remained stable on the electrodes after removing the applied voltage or even reversing the field (Supplementary Fig. 7).

**Fig. 2:**
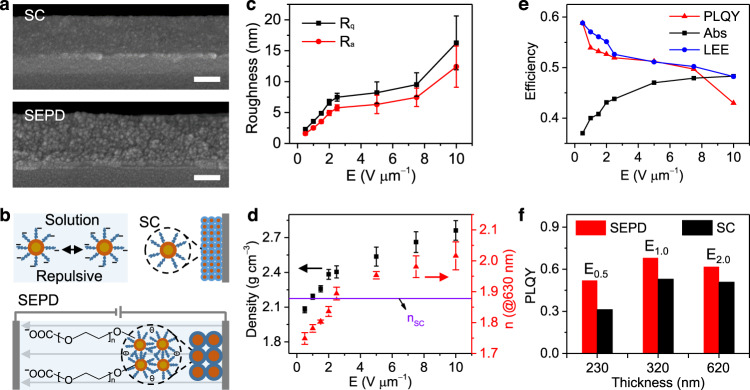
Characteristic control of QD patterns. **a** Cross-sectional SEM images of QD films prepared by spin coating (SC) and SEPD. Scale bars, 100 nm. **b** Illustration for the formation mechanism of SEPD QDs film. In polar solvents, ligands dissociate from the surface and form the repulsive interaction between particles responsible for colloidal stabilization. When the electric field is applied, charged particles are driven to move to the electrodes of opposite polarity and the repulsive forces between the particles will be overcome by the electric field. Then particle aggregation occurs due to the attractive hydrogen-bonding interaction. *R*_q_ (root mean square roughness (**c**), black line), *R*_a_ (roughness average (**c**), red line) values, density (**d**, black square), and *n*@630 nm (**d**, red triangle) of the QD films fabricated by SEPD at different electric fields (*E*). Error bars indicate standard deviations of the measured values from several samples. **e** Abs and PLQYs of QD films fabricated by SEPD at different electric fields, whose change is consistent with the simulated light extraction efficiency (LEE, blue line). **f** PLQY comparison between QD films with different thicknesses fabricated by SC and SEPD under an electric field of 0.5, 1.0, and 2.0 V μm^−1^.

Cross-section structures of QD films were further measured by high-resolution SEM to analyze the accumulation and stable deposition of QD particles on electrodes. As shown in Fig. 2a, the SEPD fabricated QD film is composed of larger particles than that of QD films prepared by spin coating (SC), indicating that the small QDs were aggregated into large particles under electric field force. Because the repulsive forces between the QDs were overcome by the electric field^28^, charged QDs were forced to get close to each other and form insoluble big particles due to the adhesion with intermolecular hydrogen bonds of PEG ligands (Fig. 2b). Therefore, deposited QDs would not be dissolved and dispersed by solvent, so they could be stably adhered on the electrodes. This stable single polarity electrode deposition characteristic effectively avoids the cross-contamination of in-plane multicolor QD patterns. In addition, the particle size increases from single QD to “oligomers” after the SEPD process, which could allow more voids in SEPD films and enhance the scattering coefficient of particles^37^. All these characters would increase the effective optical path length, resulting in the enhancement of the absorption and emission of the active material inside^38^. Thus, the QD films fabricated by SEPD may exhibit better PL performance than SC.

In EPD, the applied electric field also serves as a driving force to accelerate the charged particles towards the electrode of opposite charge^27^. With the increase of the electric field, the driving force and moving speed of particles will increase^28^, which will greatly affect the morphology and packing state of QDs films. To evaluate the effect of electric field on the SEPD films, the surface morphology, bulk density, and refractive index of QDs films deposited under different electric fields were measured. As shown in Supplementary Fig. 8, the glass cells secured 10 μm cell gap apart with parallel whole-face electrodes were used to guarantee a uniform vertical electric field so as to achieve SEPD films with uniform thickness. The same volume of QDs solution with the concentration of 50 mg mL^−1^ was injected into the glass cells. Then different electric fields in the range of 0.5–10 V μm^−1^ were applied on the cells, respectively. After SEPD, all QDs were deposited on the positive electrodes and QDs films with uniform thickness were obtained (Supplementary Fig. 8b).

Supplementary Figure 9 shows atomic force microscopy images of QDs films under different electric fields. Under a low electric field, the QDs films possess smooth surfaces. And the roughness of QD films increases with the increasing electric field (Fig. 2c) probably because of the increase of deposition rate^30^. These findings would enlighten the interface-controllable preparation for optoelectronic devices^39,40^. As shown in Supplementary Fig. 10 and Fig. 2d, with the increase of electric field force, the size of oligomer changes little, while the film thickness decreases and the packing density increases. In addition, the refractive index (*n*) associated with packing density^41^ of films can also be tuned by an electric field (Supplementary Fig. 11), such as the *n*@630 nm (Fig. 2d) can be increased from 1.749 to 2.016. While the *n*@630 nm of QD film prepared by SC is 1.878. These results further testify the packing density of SEPD film can be tuned by an electric field. Therefore, this packing density controllable characteristic would offer new strategies for tuning the charge transport of optoelectronic devices and optical gain property of lasers and gain media^41,42^.

Refractive index as a crucial factor for tailoring the light-matter interactions, plays an important role in light out-coupling efficiency of photonic and optoelectronic devices^43^. Thus, the absorption efficiency (Abs) and PLQY of these films excited by a 405 nm blue light were measured to estimate their optical properties. As shown in Fig. 2e, absorption depends strongly on the film packing density is enhanced and PLQY is adjusted from 59 to 43%, which is attributed to the change of light extraction efficiency (LEE) due to the change of refractive index (Fig. 2e and Supplementary Fig. 12), because the time-resolved PL spectra, excitation spectra and emission spectra of the QDs remain unchanged before and after SEPD (Supplementary Fig. 13). This method of adjusting the density and refractive index enables us to tailor the performance of QD devices and achieve higher luminous efficiency than conventional solution-processed methods. As shown in Fig. 2f, compared with QD films prepared by SC, SEPD fabricated films with the same thickness demonstrate even higher brightness (Supplementary Fig. 14) and exhibit higher luminance conversion efficiency due to the lower refractive index and larger scattering coefficient of SEPD film, indicating the superiority of our SEPD technique.

Besides performance, the reliability of the SEPD process and robustness of materials are required for realizing QD-based displays. We prepared ten 4 × 4 cm patterned QD films in the same way to estimate the repeatability of the SEPD process. As shown in Supplementary Fig. 15, the morphology of different films is almost the same. And the patterned QD array possesses good in-plane luminescence uniformity (>80%, Supplementary Fig. 16). To confirm the mechanical stability of QD films, we applied solvent washing using PGMEA. The results showed that the QD film was kept intact physically and the PLQY remained unchanged even after ten times of solvent washing (Supplementary Fig. 17). And the adhesion robustness of deposited QD film was verified by a cross-cut test (>3B, Supplementary Fig. 18). Moreover, the QD patterns exhibited high PLQY and an excellent stability after 1000 h of blue light aging under a light power density of 0.5 W cm^−2^ (Supplementary Fig. 19). All these results indicate good repeatability and stability of our SEPD technique, encouraging us to further construct multicolored QD patterns using spatial (place color pixels side-by-side) and tandem color (stack color pixels vertically) by simply repeating the SEPD steps.

### SEPD for full-color QD pattern arrays

The QDs can be decorated with different types of ligands to achieve either positive or negative charges. Besides the CdSe/ZnS QDs terminated with PEG-COOH, the CdSe/ZnS QDs modified with oleic acid, n-octyl mercaptan, or PEG-NH_2_, and InP/ZnS QDs treated with oleic acid, oleylamine and PEG-COOH can also be patterned by the SEPD method (Supplementary Fig. 20). Among which, CdSe/ZnS QDs terminated with PEG-NH_2_ are positively charged due to the ionization of amino ligands in an ethanol solution, which were only deposited to the negative electrodes by an electric field (Supplementary Fig. 20c). Therefore, the surface charge on QDs can be tuned by exchanging the coordinating ligands and solution. By sequentially depositing QDs with different charges, the results indicated the versatility of this approach to various kinds of QDs, which makes it very attractive for multicolor patterns via sequential SEPD.

**Fig. 3:**
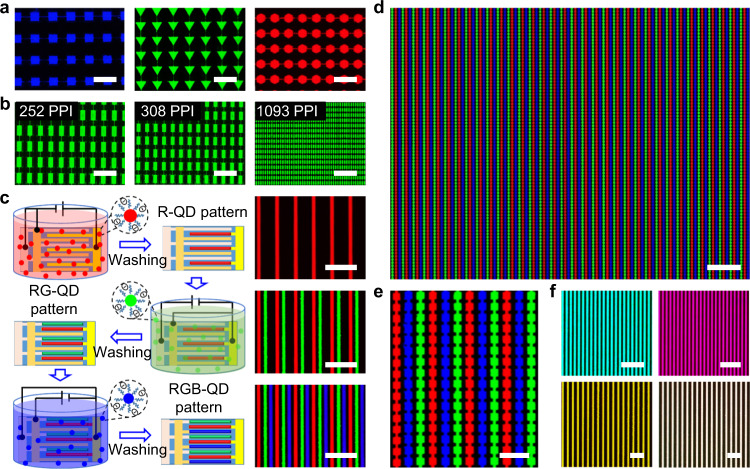
SEPD for full-color QDs pattern arrays. **a**, **b** Fluorescence images of an array of different QD patterns under UV light (blue square pattern, green triangle pattern, and red hexagon pattern). The PL images showing aligned pixels whose resolution are ranging from 252 to 1,093 PPI (Fig. 3b). Scale bars, 200 μm. **c** Schematic illustration and corresponding fluorescence images of sequential SEPD red, green, and blue QDs for fabricating full-color patterns of QDs. After each SEPD process, the substrate is washed by pure solution. Scale bars, 200 μm. **d**, **e** Fluorescence images of RGB QD patterns fabricated by three-step SEPD. Figure 3e is a magnified view of Fig. 3d. Scale bars are 200 and 50 μm, respectively. **f** PL microscopy images of multi-color and white vertical patterning fabricated by multi-step depositing different QDs on the same electrode, which demonstrates the SEPD QD films can be stacked vertically, as well as horizontally. Scale bars, 100 μm.

Afterward, we achieved green and blue QD micro-line arrays using our green- and blue-emitting QDs by the SEPD method, as shown in Supplementary Figs. 21 and 22, respectively. The fluorescent images of the green and blue QD patterns under 365 nm UV excitation are homogeneous and bright. Patterned electrodes with different shapes and sizes were designed to explore the potential of SEPD in patterning QDs. Figure 3a and Supplementary Fig. 23 demonstrate the large-area QD emitting pixel array with different shapes, sizes, and colors, which consists of tens of micron-sized pixels (triangle, rectangle, square, hexagon and circle patterns). High-resolution aligned green pixels, ranging from 252 PPI (43.9 × 90.2 μm pixel size) to 1,093 PPI (14.6 × 30.5 μm pixel size), were created by two-step depositing QDs both on positive and negative electrodes (Fig. 3b). Our results demonstrate the advantages of SEPD for arbitrarily patterned high-resolution, large-scale QD arrays, facilitating the creation of devices with the ultrahigh pixel densities required in emerging display applications (such as augmented reality and virtual reality).

Based on the above results, we demonstrated a multi-step SEPD method to construct a full-color QD pattern array. In this demonstration, QDs terminated with PEG-COOH (or PEG-NH_2_) were selected due to their stable single polarity electrode deposition characteristic. As shown in Fig. 3c, the in-plane three-electrode substrate (preparation method shown in Supplementary Fig. 24) with applied voltage was sequentially placed into the PGMEA solution containing red, green, and blue QDs (see Methods for experimental details). Then these QDs were successively deposited on the corresponding positive (negative) electrodes. Finally, the RGB pattern array was obtained (Fig. 3c). It can be seen from the fluorescent images shown in Fig. 3c that, the second SEPD process did not affect the prefabricated QD patterns due to the excellent stability of QD films. Moreover, the luminescence characteristics of underlying QD layers were not affected by solvent washing and deposition of another QD layer (Supplementary Fig. 25). In a similar way, red-green, green-blue and red-blue arrays of different shapes were successfully obtained using QDs with different color combinations (Supplementary Fig. 26). Figure 3d, e displays fluorescence images of a large-area RGB QD pattern array after three-step SEPD. The size of every RGB square pixel in the image (Fig. 3e) is 14 × 14 µm, and the spacing between two adjacent color squares is 7.0 µm. Considering Fig. 3e (consisting of 12 RGB pixels horizontally and 16 RGB pixels vertically), the resolution is about 1075 PPI (>1000 PPI). In addition to the patterns placed side-by-side, exploiting the feasibility of vertically stacking different colored QDs is interesting. Figure 3f shows a fluorescence image of cyan, magenta, yellow, and white tandem QD patterns, formed by sequentially depositing different colored QDs on the same electrode. The spatially uniform patterns of multicolor fluorescence suggest flexibility of the SEPD processes and the potential applications in constructing PL and electroluminescence (EL) layers for full-color displays.

### SEPD QLED performance

**Fig. 4:**
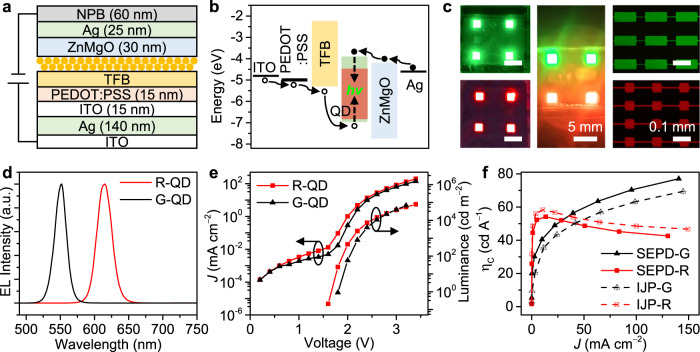
Optoelectronic properties of SEPD-QLEDs. **a** Schematic illustration of the device structure of SEPD processed QLEDs. **b** Energy band diagram of the QLEDs. **c** Images and microscopy images of SEPD G-, R-, GR-QLEDs, G-QLED pixels, and R-QLED pixels. Scale bars, 5 and 0.1 mm, respectively. **d** Normalized EL spectra of green and red SEPD QLEDs. **e** Current density−luminance−voltage (*J−L−V*) characteristics of the green and red SEPD QLEDs. **f** Current efficiency (η_C_) as a function of current density for the SEPD QLED and IJP-QLED.

Such a SEPD method not only works well with good performance for PL devices discussed above, but also shows promise in fabricating EL devices. To be compatible with the thin-film transistor technology for practical applications, a top-emitting QLED with a Fabry-Pérot cavity structure through the SEPD process was fabricated as a proof of concept to realize full-color, active QD displays. Figure 4 shows the QLED device structure (Fig. 4a) and a proposed flat band energy diagram (Fig. 4b), with energy levels taken from literature^29,44^. The device structure of QD-LED consists of silver (Ag) reflective mirror on an indium–tin oxide (ITO) glass substrate, an ITO transparent anode, a PEDOT:PSS as hole injection layer, a hole transport layers of poly(9,9-dioctylfluorene-co-N-(4-(3-methylpropyl))diphenylamine) (TFB), QDs as the emissive layer, an electron transport layers of ZnMgO nanoparticles, an Ag cathode and N,N′-bis-(1-naphyl)-N,N′-diphenyl-1,1′-biphenyl-4,4′-diamine (NPB) light extraction layer. The thickness of each layer was precisely designed and controlled based on the microcavity effect that distance between the Ag reflective surfaces (length of a cavity) is proportional to the emitting wavelength of the device.

The detailed device structures are shown in Supplementary Fig. 27. Each functional layer possesses clear and clean boundaries, and good thickness uniformity, which would help devices to achieve high performance. Figure 4c shows the EL characteristics of our G-, R-, RG-QLEDs, G-QLED pixels, and R-QLED pixels array with a common bias voltage of 2 V. Bright and uniform green, red, and green-red dual-color emission can be observed, indicating that our SEPD method is applicable to full-color QLEDs. Moreover, compared with some QLED via inkjet printing (IJP) (Supplementary Fig. 28), SEPD-QLEDs display more uniform fluorescence due to the electric field induced inhibition of the coffee-ring effect and the free of the capillary effect of bank structure (Supplementary Fig. 3). The normalized EL spectra of our G- and R-QLEDs are illustrated in Fig. 4d. With emission wavelength peaks at *λ* = 550 nm and 615 nm, the FWHMs are less than 25 nm for both colors. All devices exhibit very saturated and pure colors, as demonstrated by the Commission Internationale de l’Eclairage (CIE) chromaticity diagram shown in Supplementary Fig. 29.

The angular distribution of the SEPD-QLED shown in Supplementary Fig. 30 is almost identical to that of IJP-QLED. The emission of both QLEDs is primarily along the substrate-normal direction due to microcavity. Figure 4e shows the current density−voltage and luminance−voltage characteristics of SEPD green and red QLED. The maximum brightness of over 67,111 cd m^−2^ at 3.2 V and 79,489 cd m^−2^ at 3.4 V were achieved for green and red QLEDs, respectively. In addition, the green and red QLEDs exhibit peak current efficiency (η_C_) of 77.0 cd A^−1^ and 54.2 cd A^−1^, respectively, which are on par with the properties of the QLED prepared by IJP (Fig. 4f). An η_C_ histogram for 48 devices shows an average η_C_ of 69.1 cd A^−1^ and 47.6 cd A^−1^ for green and red QLEDs, respectively (Supplementary Fig. 31), indicating that the device performance is highly reproducible.

Furthermore, our method is compared with other patterning methods to highlight the value of the SEPD technique. Supplementary Table 2 shows a comparison of various patterning methods including inkjet printing, photolithography, contact transfer, electrohydrodynamic jet printing, as well as our SEPD. The SEPD technique demonstrates the following advantages: accurate control of thickness, low damage of QDs, easy implementation of both large area and high resolution, smooth morphology (need uniform electric field) leading to high-quality interfaces with optoelectronic devices. Thus, it can benefit large-scale devices with relatively high PPI, and can be easily integrated with photolithographic processes to achieve mass fabrication.

In summary, we have demonstrated high-resolution, large-area full-color QD emitting pixel arrays and QLED devices fabricated by selective EPD technique. The QD patterns only deposited on single polarity electrodes exhibit controllable surface morphology, packing density, and refractive index, which is adaptable for various QD optoelectronic devices to improve their performance. High-efficiency full-color PL and QLED arrays demonstrate versatile utilities of the current work. The approaches reported here represent a further step towards the application of colloidal QD devices featuring high performance, low cost, and large area. Though we used rigid glass as the substrate for demonstration, the SEPD method does not require a rigid substrate, it can extend to flexible substrates, 3D structures, and other complex structure surfaces. These results suggest the universal applicability of this simple large-area SEPD process, using various functional nanomaterials (such as QDs we demonstrated), for LEDs, displays, photovoltaics, photodetectors, and bio-imaging devices, and potentially on any substrates.

## Methods

### Materials

The blue, green, and red quantum dots used here were purchased from Mesolight Inc (Suzhou, China). Octane (extra dry, 99%) and propylene glycol monomethyl ether acetate (PGMEA, ≥99.5%) were purchased from Sigma Aldrich (Shanghai, China). Poly(9,9-dioctylfluorene-co-N-(4-(3-methylpropyl))diphenylamine) (TFB) was purchased from American Dye Source. Colloidal ZnMgO nanocrystals in an ethanol solution, PEDOT:PSS and N,N′-bis-(1-naphyl)-N,N′-diphenyl-1,1′-biphenyl-4,4′-diamine (NPB) were provided and fabricated by Guangdong Juhua Printed Display Technology Co., Ltd. Patterned ITO-coated glass cells (sheet resistance, 15 Ω sq^−1^) were purchased from Hengshang precision instruments company (Shanghai, China). Patterned ITO-coated glass substrates and the whole ITO/glass substrates whose boundary were covered with 10 μm-height photo spacer structures were fabricated by Shenzhen China Star Optoelectronics Semiconductor Display Technology Co., Ltd. All materials were used as received.

### RGB pixels fabrication

Figure 3c shows the process of fabricating RGB pixels by a three-step SEPD method. In this experiment, the red CdSe/ZnS, green CdSe/ZnS, and blue CdZnSe/ZnS QDs were all capped with PEG-COOH in PGMEA solution to make them negatively charged. Firstly, the in-plane three-electrode substrate was immersed into the red QDs (10 mg mL^−1^). One group of electrodes was positively charged with an electric field (*E*) of 5 V μm^−1^ and the other two groups were negatively charged. After about 30 s, the substrate was lifted out of the QD solution and washed by dipping into pure PGMEA solution for 10 s. Subsequently, by alternating the positively charged electrodes, green and blue QD layers were subsequently deposited with the same process (*E* = 5 V μm^−1^ and 10 mg mL^−1^ for green, and 7.5 V μm^−1^ and 20 mg mL^−1^ for blue QDs). The blue QDs demand a higher driving field and concentration to obtain thicker film because of their lower luminous efficiency. After the same deposition and cleaning time, drying in an oven at 80 °C for 20 min, at last, the RGB pixels were obtained.

### Device fabrication

The ITO/Ag/ITO substrates were first cleaned in ultrasonic detergent for 30 min and sprayed with deionized water, followed by soaking in ultrasonic deionized water for 15 min and baking in an oven for 30 min. Then they were cleaned with an oxygen plasma cleaner for 5 min. The substrates were then transferred into a N_2_-filled glove box for the preparation of layers of PEDOT: PSS, TFB, and QDs and ZnMgO nanoparticles. A 15 nm thick PEDOT: PSS and TFB layers were spin-coated followed by baking at 150 °C for 20 min. In accordance with the microcavity effect, the optimized TFB layer thickness is different for specific color-emitting QLEDs and is proportional to the emitting wavelength of a device. The green QLEDs, therefore, have the optimized TFB layer thickness of ∼30 nm (8 mg mL^−1^ TFB in chlorobenzene solution, 3,000 r.p.m. spin speed), while those for red QLEDs are ∼50 nm (TFB, 20 mg mL^−1^, 3,000 r.p.m.), respectively. A TFB/PEDOT: PSS-on-ITO/Ag/ITO/glass substrate and a whole ITO/glass substrate whose boundary was covered with 10 μm-height photo spacer structures were secured together with their conductive sides parallel and facing toward one another. An electric field of 10 V μm^−1^ is then applied, and the electrodes are placed into the CdSe/ZnS QDs modified by carboxylic acid in octane solution. The optimized QD layer thicknesses were ∼12 nm for red (8 mg mL^−1^ QD octane solution) and ∼10 nm for green (5 mg mL^−1^) as determined from an efficiency comparison of devices with various QD layer thicknesses. After a fixed time (a few minutes in duration), the electrodes are lifted out of the QD solution and, with the voltage bias remaining on, washed by dipping into a vial filled with neat octane for 10 s. Then the substrates were dried in an oven at 80 °C for 5 min. Then 30 nm thick ZnMgO nanocrystals (in ethanol 30 mg mL^−1^) were spin-coated onto the coated substrates at 4,000 r.p.m. for 30 s and baked at 80 °C for 10 min. Then transferred into a vacuum chamber (with a base pressure of 10^−6^ Torr), where a 25 nm thick Ag cathode and a 60 nm thick NPB were deposited by sequential thermal evaporation through a shadow mask. The devices were encapsulated in the glove box by the cover glasses using ultraviolet-curable resin.

### Characterizations

The morphology of the as-prepared QD films was examined by scanning electron microscope (SEM, ZEISS GEMINI 300), 3D optical microscope (VK-X1100), and atomic force microscopy (AFM, Nano Man VS). The cross-sectional scanning transmission electron microscope images of QLED were measured by FEI Helious G4 UC. Bright-field and fluorescence microscopy images were acquired using an inverted fluorescence microscope (Nikon, Ti-U) under the excitation of a mercury lamp (330−380 nm). The electrophoretic mobility and zeta potential of the QD solution were measured by Brookhaven ZetaPALS zeta potentials analyzer. The refractive index of QD films was measured by spectroscopic ellipsometry (J.A. Woollam RC2). The time-resolved PL spectra were recorded using a fluorescence lifetime spectrometer (Fluo Time 300). Photoluminescence quantum yields (PLQYs) were measured by using an integrating sphere on a HAMAMATSU absolute PL quantum yield spectrometer C11347. Excitation and fluorescence spectra were registered at room temperature on a Lumina fluorescence spectrophotometer. The current density−voltage−luminance (*J*−*V*−*L*) characteristics and the angular dependence of emission intensity of the devices were measured by a dual-channel Keithley 2614B source meter unit and a calibrated PIN-25D silicon photodiode.

## Supplementary information

Supplementary Information

Description of Additional Supplementary Files

Supplementary Movie 1

## Data Availability

The data that support the findings of this study are available from the corresponding author upon reasonable request.
