# Supplementary material for: Large-area patterning of full-color quantum dot arrays beyond 1000 pixels per inch by selective electrophoretic deposition
Source: Nat Commun. 2021 Jul 29;12:4603. doi: 10.1038/s41467-021-24931-x (PMC8322170; doi:10.1038/s41467-021-24931-x)
Supplement: Supplementary file 1 — Supplementary Information [file 41467_2021_24931_MOESM1_ESM.pdf]

Supplementary Information

Large-Area Patterning of Full-Color Quantum Dot Arrays Beyond 1000 Pixels Per Inch by Selective Electrophoretic Deposition

Jinyang Zhao[1,2,3], Lixuan Chen[1,2,3*], Dongze Li[3], Zhiqing Shi[3], Pai Liu[1], Zhenlei Yao[4], Hongcheng Yang[1,5], Taoyu Zou[2], Bin Zhao[3], Xin Zhang[3], Hang Zhou[2], Yixing Yang[4], Weiran Cao[3,4*], Xiaolin Yan[4], Shengdong Zhang[2*], and Xiao Wei Sun[1,5*]

[1]Key Laboratory of Energy Conversion and Storage Technologies, Ministry of Education, and Department of Electrical and Electronic Engineering, Southern University of Science and Technology, Shenzhen, Guangdong 518055, China

[2]School of Electronic and Computer Engineering, Peking University, Shenzhen, Guangdong 518055, China

[3]Shenzhen China Star Optoelectronics Semiconductor Display Technology Co., Ltd. Shenzhen, Guangdong 518132, China

[4]TCL Corporate Research, 1001 Zhongshan Park Road, Nanshan District, Shenzhen 518067, China

[5]Shenzhen Planck Innovation Technologies Co. Ltd, Shenzhen, Guangdong 518112, China

Supplementary Note 1. Properties of QD materials

Supplementary Table 1 shows PLQY and zeta potentials of four QDs modified by PEG-COOH ligands with different contents. The absolute value of zeta potential is increased with the increase of ligand content, which indicates that the charges on QDs mainly originate from ligand ionization and can be tuned by modifying the ligand. However, ligand modification influences PLQY of QD slightly, so the QD with the highest PLQY was selected as the model compound for the construction of patterned structures. The PL spectrum of red colloidal CdSe/ZnS core/shell QDs terminated with PEG-COOH is shown in **Supplementary Fig. 1**.

CdSe/ZnS (PEG-COOH) in PGMEA				
Name	QD-1	QD-2	QD-3	QD-4
QD concentration	100 mg/mL	100 mg/mL	100 mg/mL	100 mg/mL
Solvent (PGMEA)	89.70 wt%	90.00 wt%	90.20 wt%	90.40 wt%
Ligands	**3.16 wt%**	**3.58 wt%**	**4.08 wt%**	**4.40 wt%**
CdSe/ZnS	7.14 wt%	6.42 wt%	5.72 wt%	5.20 wt%
PLQY @450 nm	79%	**91%**	73%	46%
Zeta potential (mV)	-21.01	-17.07	-38.01	-46.18
	-27.52	-37.46	-38.18	-38.97
	-24.07	-31.07	-51.77	-27.27
	-16.38	-22.61	-28.61	-64.24
	-10.72	-25.35	-19.93	-41.15
	-11.10	-28.29	-33.08	-49.31
	-22.82	-34.14	-19.37	-37.26
	-19.56	-33.01	-42.72	-43.98
	-11.21	-18.72	-46.45	-56.34
	-15.94	-21.25	-23.56	-49.62
Average (mV)	**-18.03**	**-26.90**	**-34.17**	**-45.43**
Electrophoretic mobility (μm cm s^{-1} V^{-1})	**-0.121**	**-0.180**	**-0.228**	**-0.304**

Supplementary Table 1. The zeta potentials of QDs capped with different ligand contents.

The surfaces of QDs are negatively charged by the ionized carboxylic acid due to the ligand ionization in PGMEA solution, which can be tested by zeta potential measurement. The electrophoretic mobility and zeta potential of the QD solution were measured by a phase analysis light scattering zeta potential analyzer. Supplementary Table 1 shows the zeta potentials of QDs capped with different ligand contents for ten runs. In can be seen that the average value of zeta potentials is decreased from -18.03 mV to -45.43 mV with the increase of ligand content from 3.16 wt% to 4.40 wt%. This indicates that the surface charges of QD are mainly generated by ligand ionization and can be tuned by modifying the ligand.

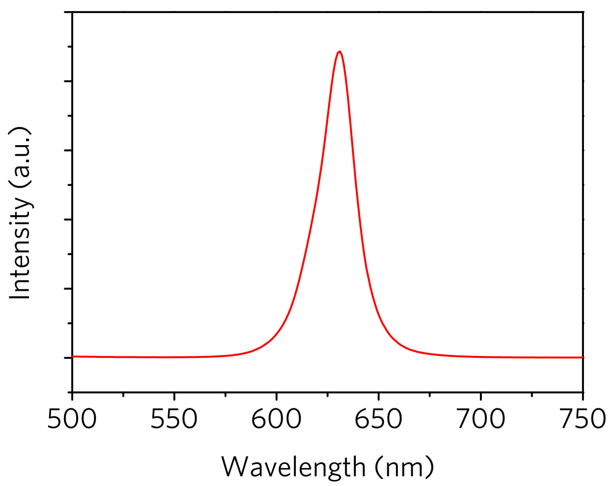

Supplementary Figure 1. PL spectrum of red colloidal CdSe/ZnS core/shell QDs terminated with PEG-COOH.

Supplementary Note 2. Morphology and size of QD pattern

The 3D OM images (**Supplementary Fig. 2**) and cross-sectional SEM image (**Supplementary Fig. 3**) of deposited QD stripe pattern show that these deposited QD microstructures have close-packed arc cross-section structure without visible pinhole or surface defects, and the planeness of QDs patterns can be tuned by changing their thickness and linewidth. With enough QDs in solution and for fixed deposition time, the film thickness monotonically increases with the electric field and QD concentration (**Supplementary Fig. 4**). The linewidth and space of QD pattern mainly depend on the designed electrode (**Supplementary Fig. 5**). The width of QD pattern can be reduced to about 2 μm by using 2 μm-width electrode (**Supplementary Fig. 6**).

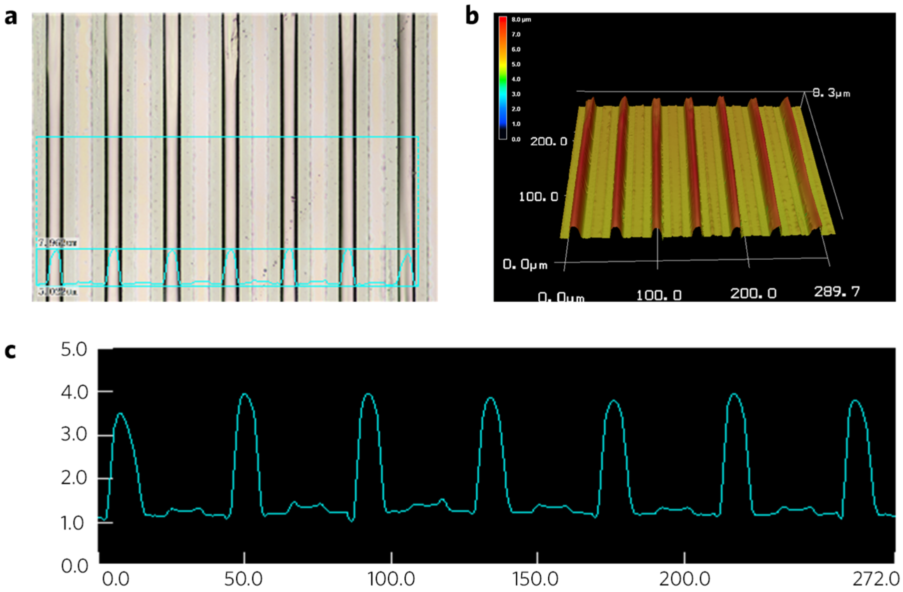

Supplementary Figure 2. The 3D OM images of deposited QD pattern. **a,** Bright field image, **b,** 3D OM image, and **c,** corresponding cross-sectional profile of the fabricated QD pattern.

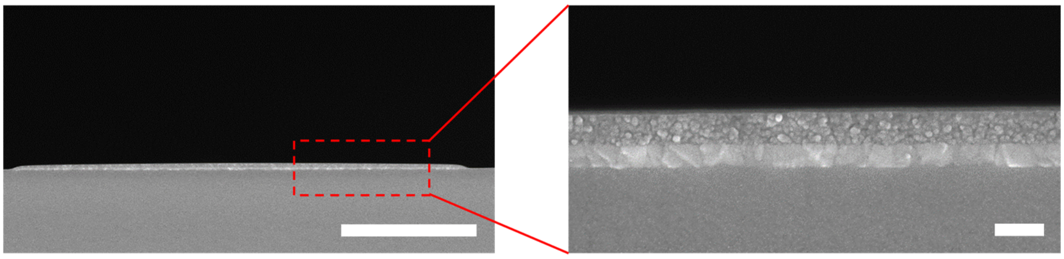

Supplementary Figure 3. Cross-sectional SEM image and corresponding enlarged image of QD stripe with thickness of 0.1 μm, showing the uniform thickness of SEPD pattern. Scale bars, 5 μm and 200 nm, respectively.

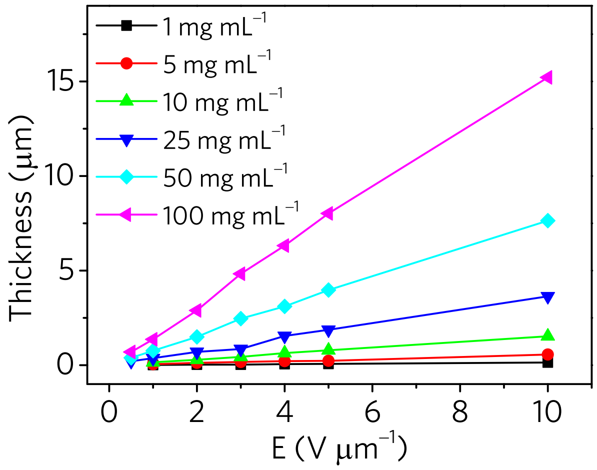

Supplementary Figure 4. Deposited QD stripe layer thickness versus the electric field at various QD concentration.

Supplementary Figure 4 and Figure 1j demonstrate the thickness of QD patterns as a function of electric field at various QD concentration. As the supply of QDs and same deposition duration are sufficient, the thickness monotonically increases with the electric field and QD concentration. The curve has a linear slope of about 1.58×10^{-17} $m^5\ g^{-1}\ V^{-1}$. Therefore, the thickness of QD patterns can be precisely controlled through a combination of electric field and QD concentration.

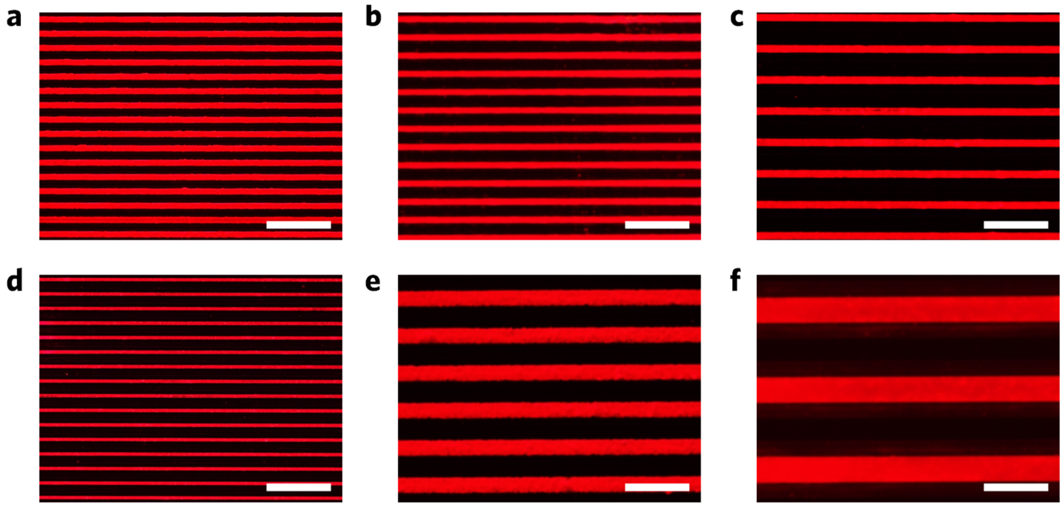

Supplementary Figure 5. Fluorescence images of QD stripe array with different linewidth and space. All scale bars, 100 μm.

As shown in Supplementary Fig. 5, it demonstrated the QD microscale line array with the different width and different spacing. The base linewidths/spaces of these stripes were 10.1/14.9, 10.0/19.8, 10.0/38.7, 5.0/17.6, 24.7/34.7 and 41.2/82.9 μm, respectively. SEPD yields high-resolution arrays of QD with line width of ≈ 5 μm as shown in Supplementary Fig. 5d, which could match the ultra-high-resolution requirement for display.

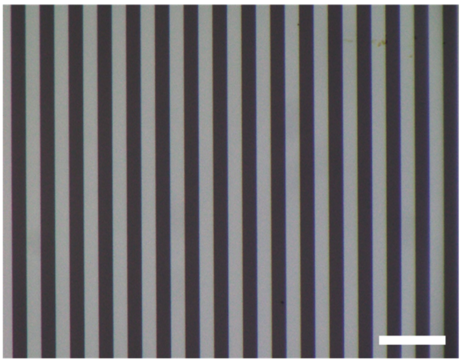

Supplementary Figure 6. Bright field image of patterned ITO electrodes prepared by photolithography. Scale bar, 10 μm.

In our experiment, we have tested electrode width down to 2 μm (Supplementary Fig. 6). As a result, the QD stripe width obtained in our case is about 2.06 - 2.10 μm (close to the ITO width of 2 μm) by carefully measurement (Fig. 1k). The width error is less than 5%.

Supplementary Note 3. Characteristic control of QD patterns

The QDs could stably deposited on the electrode after removing the applied voltage or even reversing the field (**Supplementary Fig. 7**), because the QDs modified by PEG-COOH were forced to flocculate due to the adhesion of PEG molecules and form a solid packing structure. SEPD QDs films with uniform thickness were obtained by using uniform vertical electric field (**Supplementary Fig. 8**), which provides a convenient platform for estimating their characteristic. Thus, based on these films, the surface morphology (**Supplementary Fig. 9**), bulk density (**Supplementary Fig. 10**) and refractive index (**Supplementary Fig. 11**) of QDs films deposited under different electric fields were measured. The roughness of QD films increases with the increasing the electric field probably because of the increase of deposition rate. The packing density and refractive index increases the increase of electric field. Simulation results in **Supplementary Fig. 12** show that the light extraction efficiency of QD film is decreased with the increase of refractive index. The time-resolved PL spectra, excitation spectra and emission spectra of the QDs remain unchanged before and after SEPD (**Supplementary Fig. 13**). All these results indicate that absorption efficiency and PLQY of these SEPD QD films are changed with the electric field due to the change of refractive index. Therefore, SEPD fabricated films with the same thickness demonstrate even higher brightness (**Supplementary Fig. 14**) due to the lower refractive index and larger scattering coefficient of SEPD film, indicating the superiority of our SEPD technique. In addition, SEPD process has good repeatability and stability, the morphology of different films is almost the same (**Supplementary Fig. 15**) and patterned QD array

possesses good in-plane luminescence uniformity (**Supplementary Fig. 16**). The SEPD QD films possess good mechanical stability (**Supplementary Fig. 17**), adhesion (**Supplementary Fig. 18**) and optical stability (**Supplementary Fig. 19**).

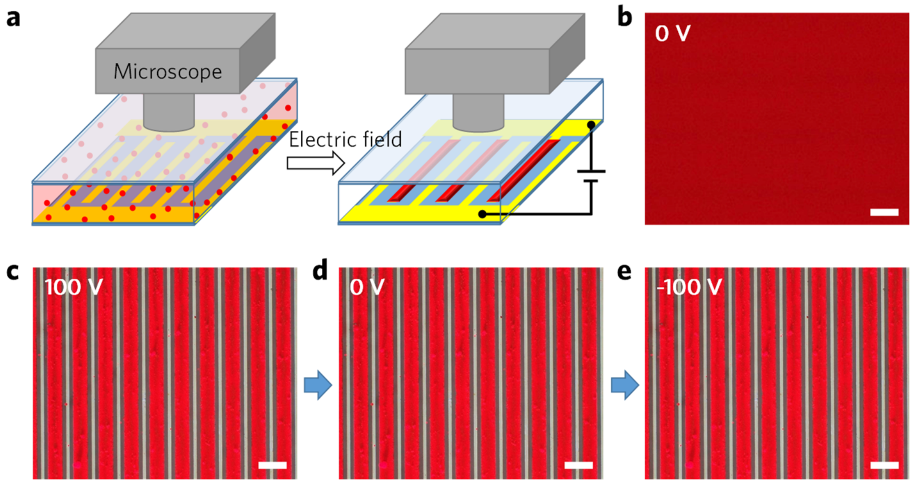

Supplementary Figure 7. Stable single polarity electrode deposition. **a,** Schematic that outlines the SEPD approach for fabricating QDs patterns via patterned electrodes in airtight glass cell. **b-e,** The PL images of the QD solution in glass cell under different electric filed. Scale bars, 50 μm.

To directly observe the movement and deposition of QDs in solution under electric field, a micro-PL system was set up as shown in Supplementary Fig. 7a. A glass cell with in-plane interdigital electrodes substrate was used as container to hold the QD solution. Red QD in PEGMA solution was injected into electrodes cell so as to cover the entire patterned electrodes area, which can be confirmed by the uniform red fluorescence in Supplementary Fig. 7b. When electric field of 5 V μm^{-1} is applied between the interdigital electrodes, the QDs whose surfaces are negatively charged were deposited to the positive electrodes (Supplementary Fig. 7c). Then remove the applied voltage, the QDs still deposited on the positive electrodes (Supplementary Fig. 7d). Even though reversing field, the deposited QDs remain no change (Supplementary Fig. 7e). All these results indicate that the QDs could stably deposited on the electrode after SEPD process,

because the QDs modified by PEG-COOH were forced to flocculate due to the adhesion of PEG molecules and form a solid packing structure.

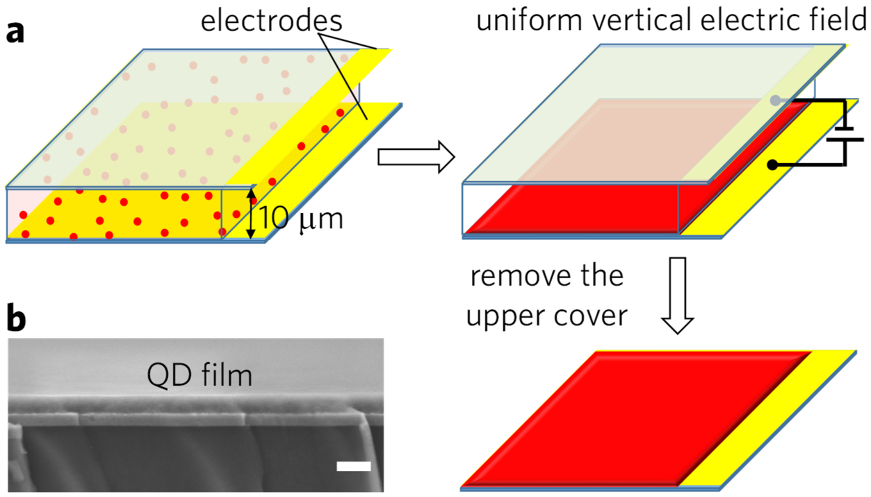

Supplementary Figure 8. SEPD QD films with uniform thickness via uniform vertical electric field. **a,** Schematic illustration of SEPD QD film with uniform thickness by using the glass cells with parallel whole-face electrodes. **b,** Cross-sectional SEM images of QD films shows the uniform thickness. Scale bar, 200 nm.

As shown in Supplementary Fig. 8a, same volume of QDs solution with the concentration of 50 mg mL^{-1} were injected into the glass cells with parallel whole-face electrodes. Then an electric field in the range of 0.5-10 V µm^{-1} is applied. After SEPD, all QDs were deposited on the positive electrodes and QDs films with uniform thickness were obtained (Supplementary Fig. 8b).

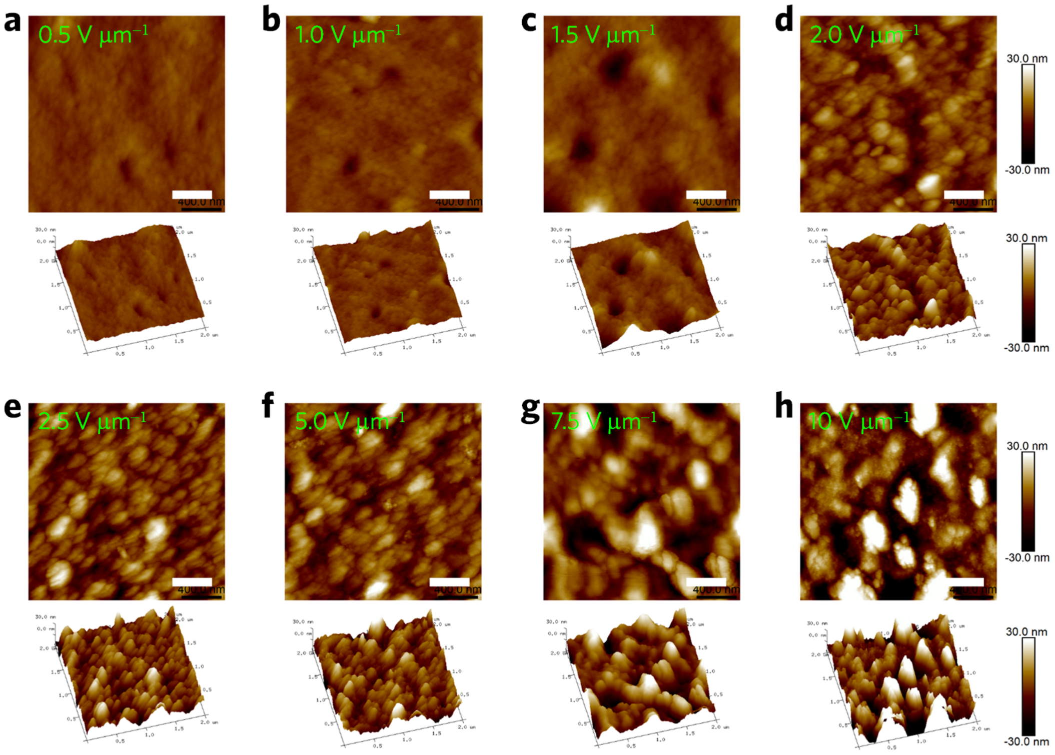

Supplementary Figure 9. Atomic force microscopy images of SEPD QD films at different electric fields. Scale bar, 400 nm.

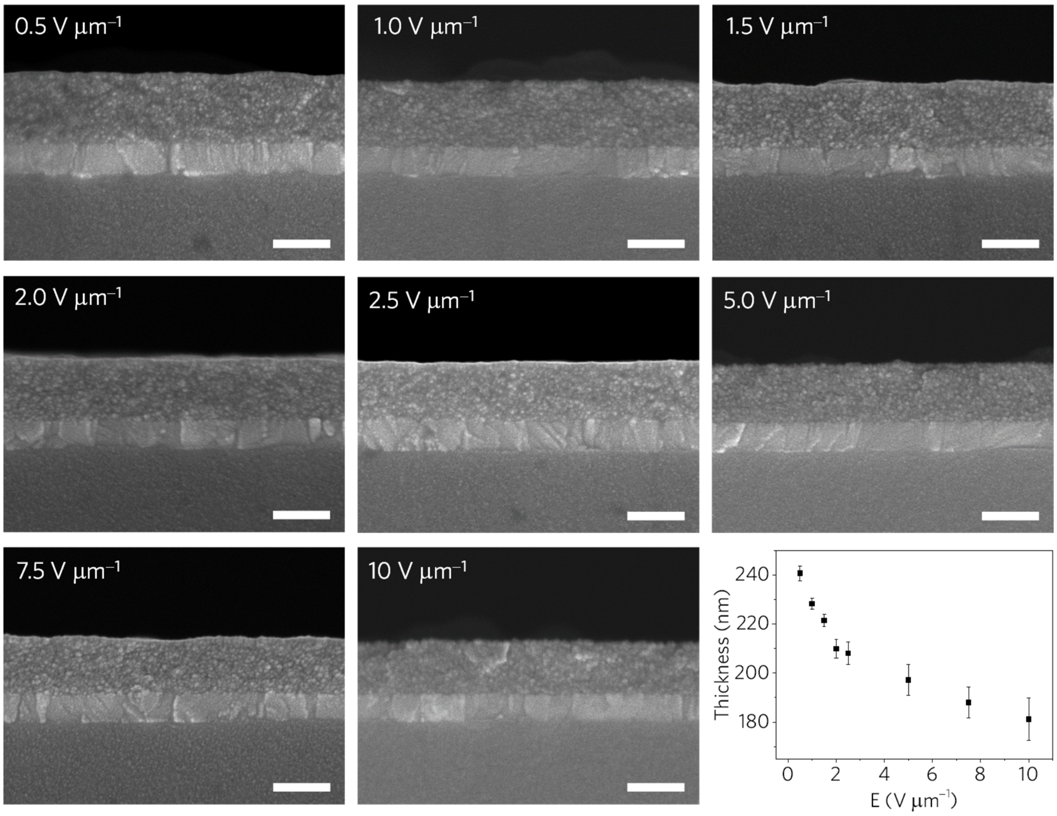

Supplementary Figure 10. SEM images and the thickness of QD films fabricated by SEPD at different electric fields. The thickness decreases with the increase of electric field. Error bars indicate standard deviations of the measured values from several samples. Scale bar, 200 nm.

The uniform QD films were fabricated as the method shown in Supplementary Fig. 8a. The density of these films can be calculated by formula: $\rho = (C_{QD} \times A \times H)/(A \times h)$. Here, C_{QD} is the QD concentration (50 mg mL^{-1}), A is the electrode area, H is the cell gap of glass cell (10 μm), h is the thickness of QD film (Supplementary Fig. 10). Therefore, the calculated densities are shown in Fig. 2d.

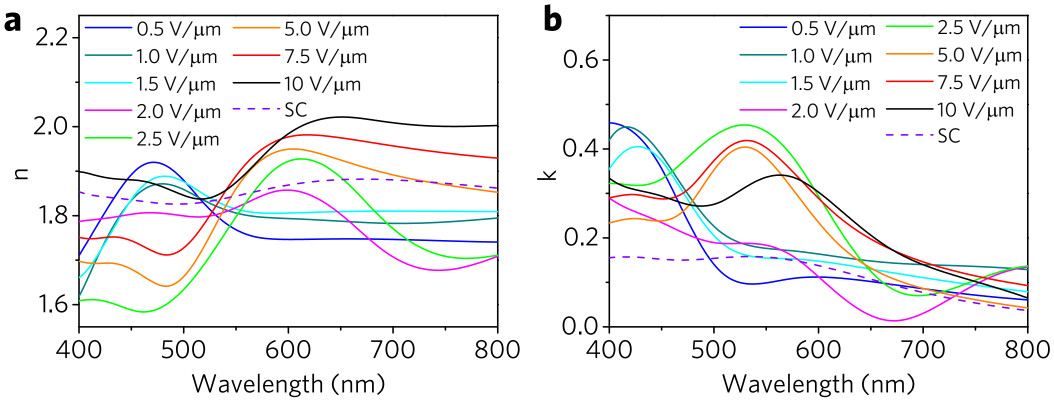

Supplementary Figure 11. The real (**a**) and imaginary (**b**) effective refractive index of the of QD films fabricated by SEPD at different electric fields and spin coating (SC).

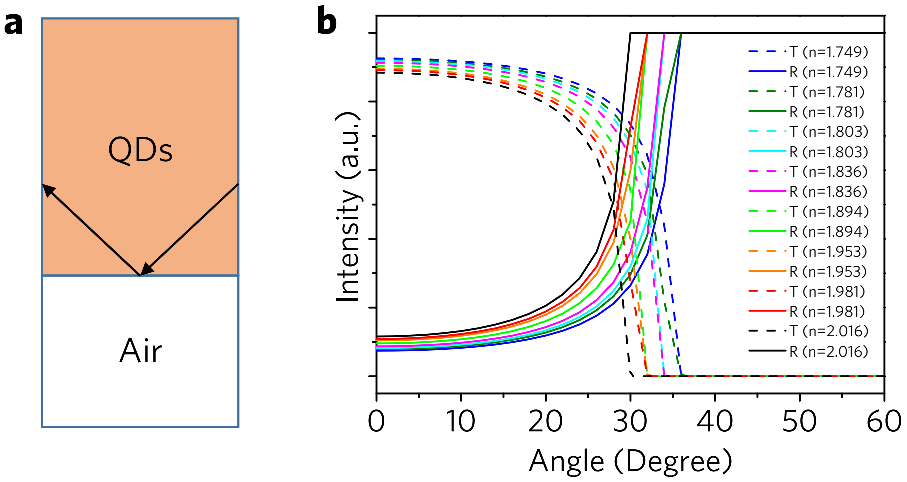

c

E ($V\ \mu m^{-1}$)	n_{QD} (@630nm)	T_{total}	normalized light extraction efficiency
0.5	1.749	15.178	1.000
1	1.781	14.743	0.971
1.5	1.803	14.367	0.947
2	1.836	14.096	0.929
2.5	1.894	13.298	0.876
5	1.953	12.868	0.848
7.5	1.981	12.596	0.830
10	2.016	12.033	0.793

Supplementary Figure 12. Simulated light extraction efficiencies of QD films with different refractive indexes. **a,** Computational model that calculates the optical properties of QD film. **b,** The transmittance and reflectance of light at various angles of incidence. **c,** The n@630 nm, simulated total transmittance (T_{total}) and normalized light extraction efficiency of QD films fabricated by SEPD at different electric fields.

Finite-element (FEM) electromagnetic simulations are performed by COMSOL Multiphysics. A thin uniform layer of QDs is setting on top of the air (Supplementary Fig. 12a). The height of QD layer is 1050 nm and the width is 50 nm with Floquet

periodic boundary conditions on the left and right sides. When incident light passes through the interface of QDs layer and air layer, part of the electromagnetic field is completely reflected because of high refractive index of QDs. Without considering absorption, we calculate the transmittance of different incident angle (Supplementary Fig. 12b) and thus the total energy of emergent light by assuming QDs are similar to perfect point light source. Then total transmittance (T_{total}) from all angle is calculated as the light extraction efficiency of QD film, which is decreased with the increase of refractive index.

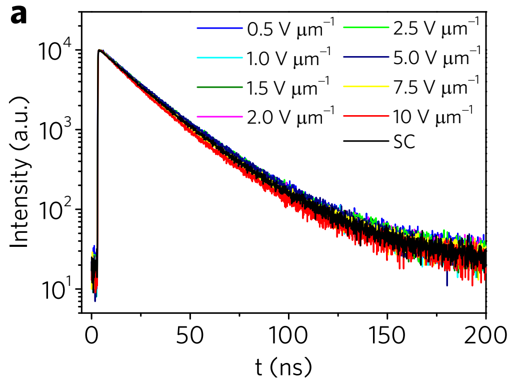

b

V µm^{-1}	τ_1 (ns)	τ_2 (ns)
0.5	23.27	21.29
1	22.94	20.95
1.5	22.87	20.95
2	23.49	21.42
2.5	23.71	21.66
5	23.97	21.94
7.5	22.50	20.50
10	21.59	19.56
SC	23.23	21.18

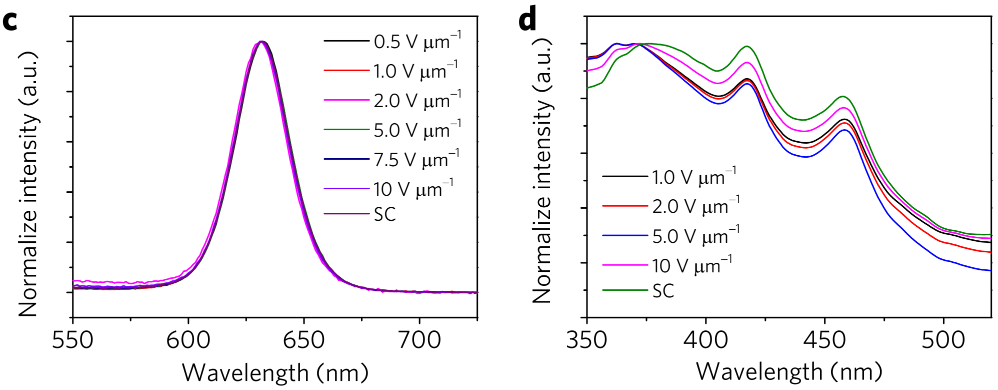

Supplementary Figure 13. Optical characteristics of QD film fabricated by SEPD and SC. Time-resolved PL spectra (**a**), fluorescence lifetime (**b**), normalize PL spectra (**c**) and normalize excitation spectra (**d**) of QD films prepared by SEPD at different electric fields and SC, respectively. All these optical properties are almost the same, indicating QDs themselves not be changed by SEPD.

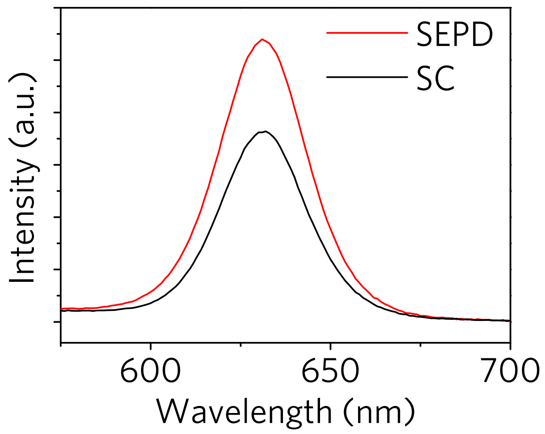

Supplementary Figure 14. Comparison of PL properties of QD films prepared by SC and SEPD. The spectra of QD films with the thickness of 230 nm fabricated by SEPD (red line) and SC (black line) excited by same blue light, respectively.

Supplementary Figure 14 compares the PL luminance of SEPD-QD film and SC-QD film. It can be seen that the PL luminance of SEPD-QD film were greater than that of SC-QD film at the same thickness. Because the particle size increases from single QD to the multimer due to the aggregation of QD particle with SEPD process, which results increasement of scattering coefficient of QDs[1]. Multiple scattering increases the effective optical path length, resulting in the enhancement of the absorption and emission of an active material inside[2]. Therefore, with larger multimer and lower refractive index of QD film, light conversion efficiency of QD film fabricated by SEPD is largely improved. These results indicate SEPD method could effectively guarantee the luminous efficiency of QDs.

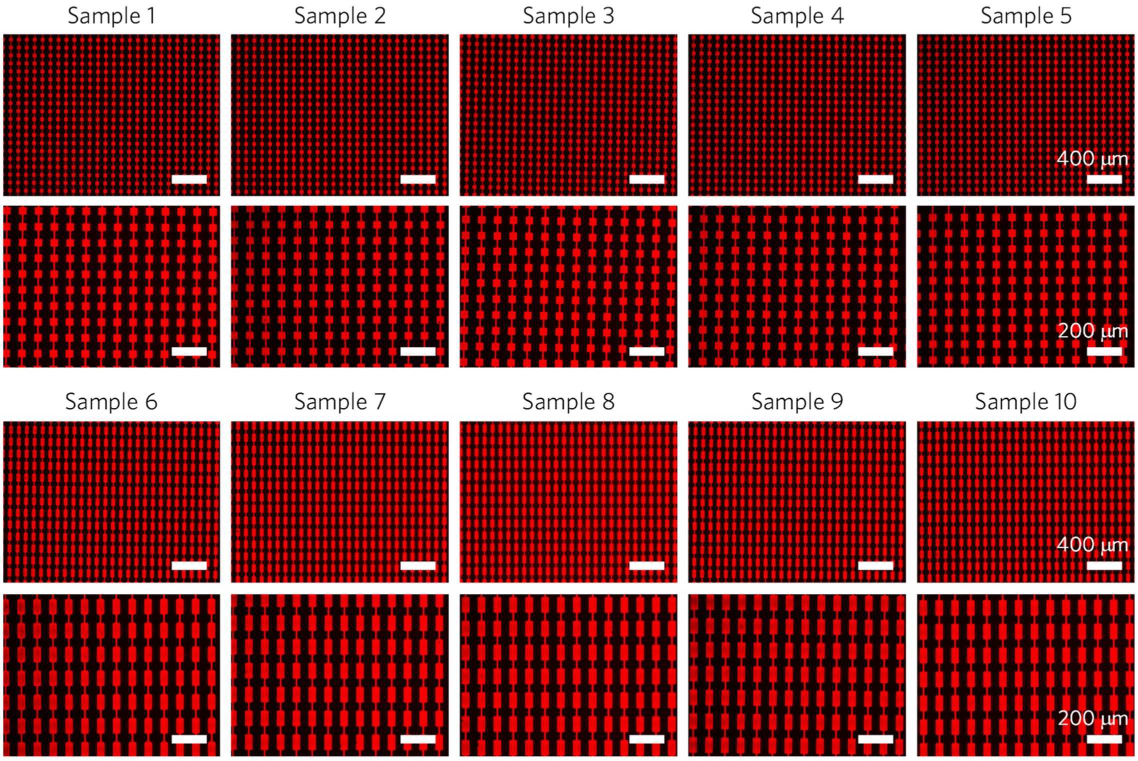

Supplementary Figure 15. PL images of the ten 4 × 4 cm QD pattern arrays fabricated under the same conditions. Five of them are square patterns and the other five are rectangular patterns. The morphology of different samples is almost consistent, indicating a good repeatability and reproducibility of our SEPD method.

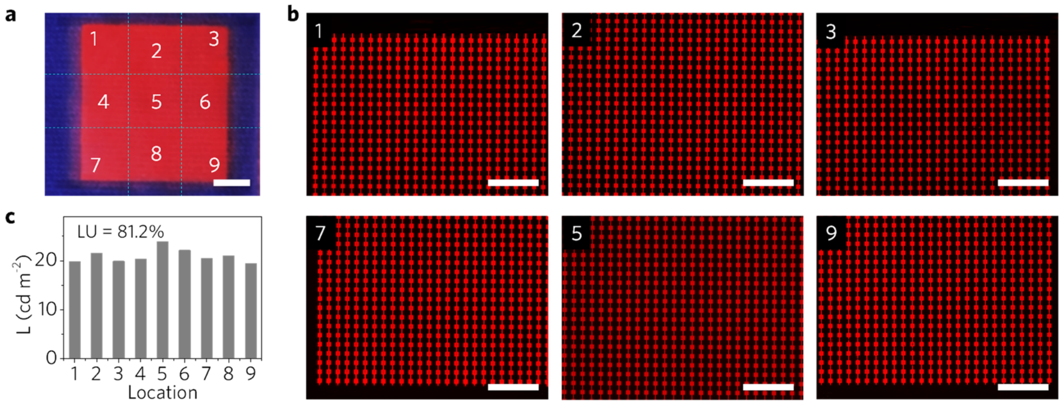

Supplementary Figure 16. a, Photograph of a 4 × 4 cm red QD pattern film. Scale bar, 1 cm. **b, c,** PL images and luminance (L) of red pattern array collected from different locations marked by the number shown in (**a**). All scale bars, 500 μm. Luminance uniformity (LU) = min(L)/max(L) = 81.2%.

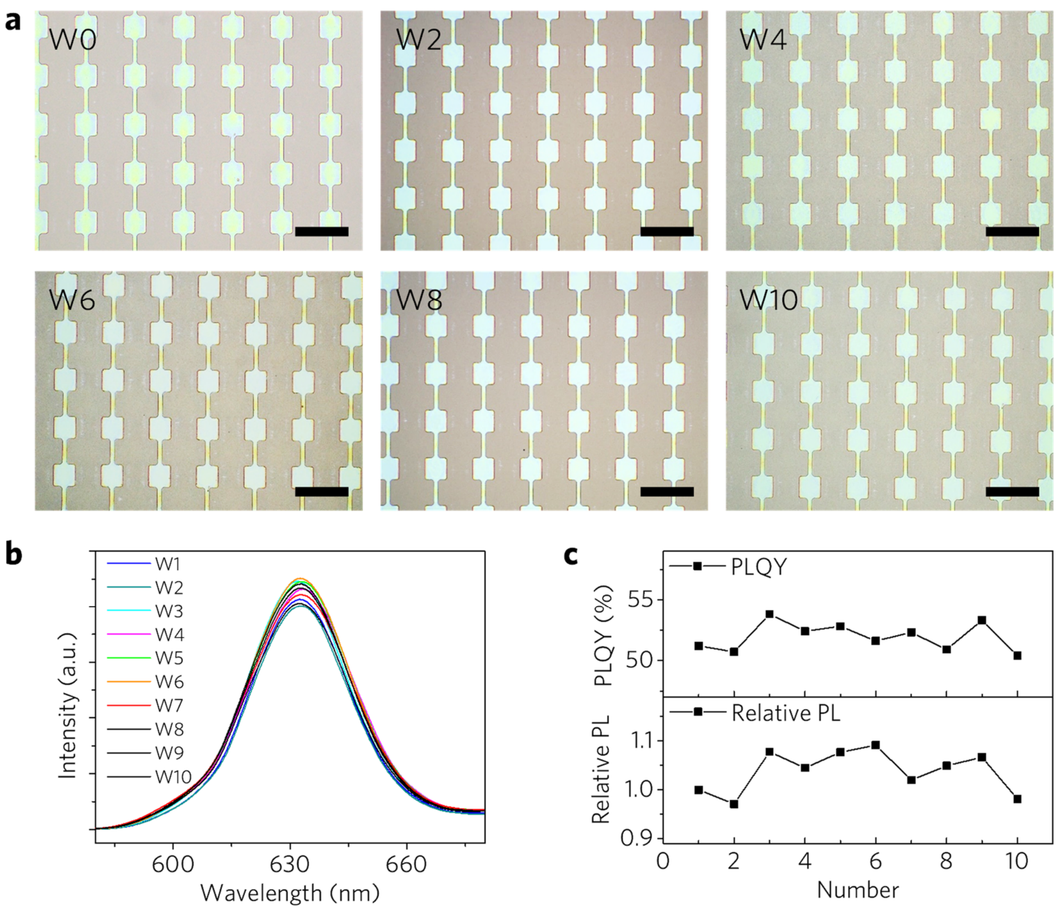

Supplementary Figure 17. a, OM images of QD patterned film before (W0) and after being washed 2 times (W2), 4 times (W4), 6 times (W6), 8 times (W8) and 10 times (W10) with PGMEA, respectively. Scale bars, 100 μm. **b, c,** PL spectra, relative PL intensity and PLQY of red patterned film as a function of the number of washing cycles. It can be seen that the QD patterned film still keeps the appearance as it is after many times of washing, manifesting a good stability against solution.

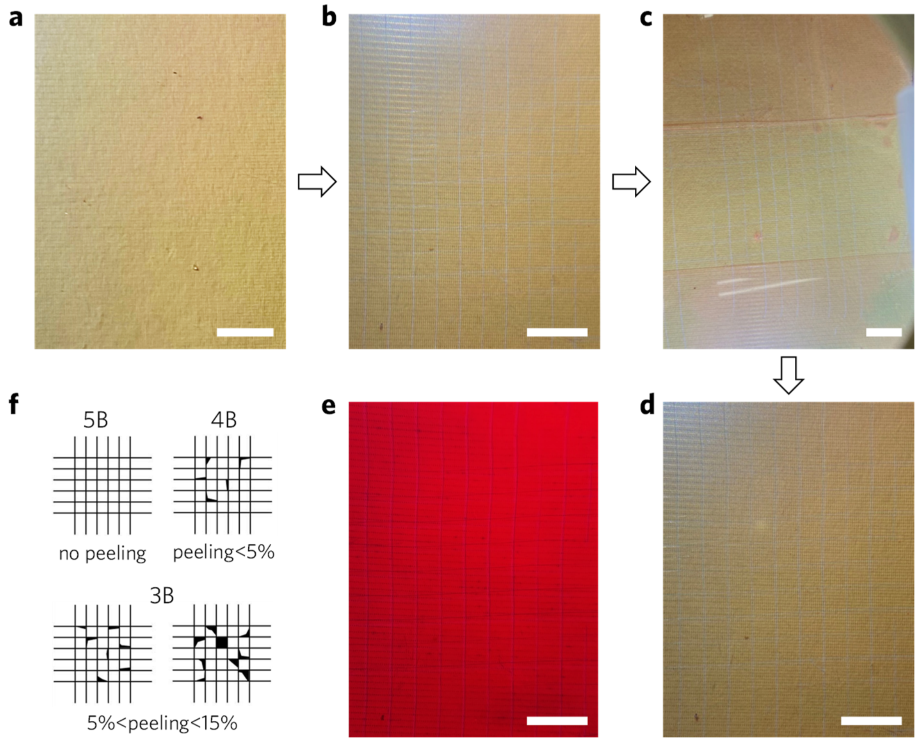

Supplementary Figure 18. Adhesion cross cut test. Photographs of SEPD QD film before cross-cutting (**a**), after cross-cutting (**b**), after being pasted with tape (**c**) and after removing with tape (**d, e**). Scale bars, 5 mm. **f,** The standard of adhesion (ASTM D3359 Method B).

The cross-cut test is a simple and easily practicable method for evaluating the adhesion of films. Typical test method is as follows: Make a lattice pattern in the film (cutting to the substrate) with the appropriate tool (Supplementary Fig. 18b). Brush in diagonal direction 5 times using a brush pen, then tape over the cut (Fig. S18c). Remove tape and examine the grid area with an illuminated magnifier (Supplementary Fig. 18d and e). A cross-cut area is not significantly greater than 5 %. Based on the standard of adhesion shown in Supplementary Fig. 18f, the result could be roughly rated as >3B, which shows that QD film has good adhesion with the substrate.

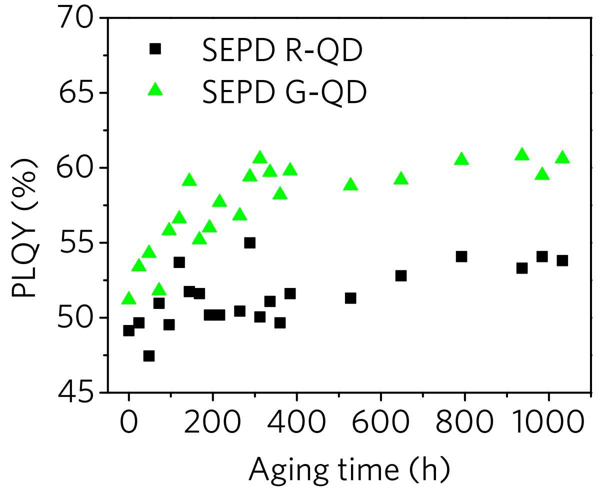

Supplementary Figure 19. The change of PLQY of SEPD red and green QD films under continuous blue light irradiation (480 nm, 0.5 W cm^{-2}).

Supplementary Note 4. SEPD for full-color QD pattern arrays

The QDs modified with different ligands in different solution can also be patterned by SEPD method (**Supplementary Fig. 20**), indicating the versatility of this approach to various kinds of QDs. Afterwards, green micro-line arrays (**Supplementary Fig. 21**), blue micro-line arrays (**Supplementary Fig. 22**) and large-area QD emitting pixel array with different shapes, sizes and colors (**Supplementary Fig. 23**) were achieved by the SEPD method. To fabricate RGB QD pattern, in-plane three-electrode substrates were prepared (**Supplementary Fig. 24**). It can be seen from the fluorescent images and spectra shown in **Supplementary Fig. 25**, luminescence characteristics of underlying QD layers were not affected by deposition of another QD layer. Therefore, red-green, green-blue and red-blue arrays of different shapes can be successfully obtained using QDs with different color combinations (**Supplementary Fig. 26**)

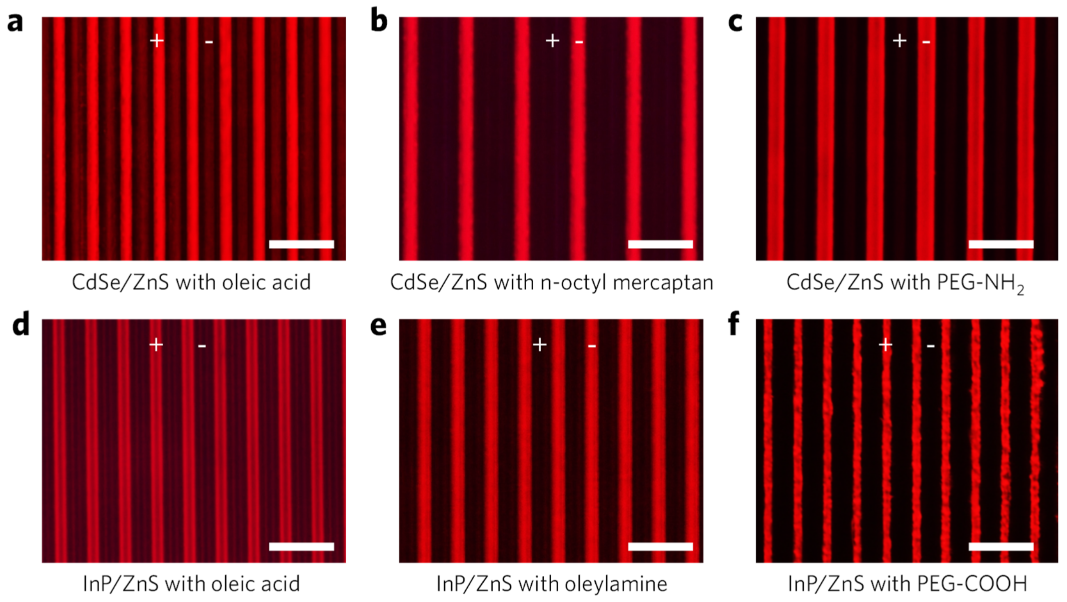

Supplementary Figure 20. PL images of SEPD fabricated stripe array using different QDs. **a,** CdSe/ZnS QDs terminated with oleic acid in octane solution, **b,** CdSe/ZnS QDs terminated with n-octyl mercaptan in octane solution, **c,** CdSe/ZnS QDs terminated with PEG-NH_2 in ethanol solution, **d,** InP/ZnS QDs terminated with oleic acid in octane solution, **e,** InP/ZnS QDs terminated with oleylamine in octane solution, **f,** InP/ZnS QDs terminated with PEG-COOH in PGMEA solution. All scale bars, 50 μm.

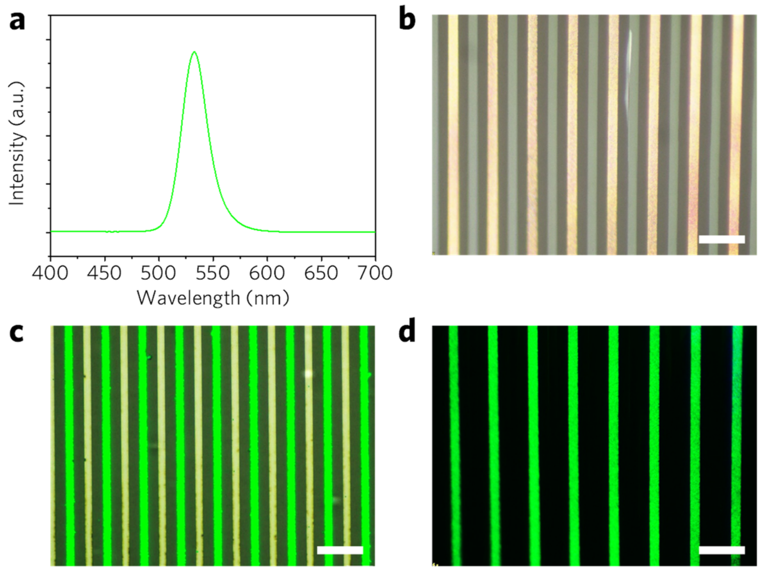

Supplementary Figure 21. Patterning of green QDs array via SEPD. **a,** PL spectrum of green CdSe/ZnS QDs terminated with PEG-COOH. **b-d,** OM images of green QD pattern irradiated by white light (**b**), white light and ultraviolet light (**c**) and ultraviolet light (**d**). These results indicate these QDs are only deposited on positive electrodes. All scale bars, 50 μm.

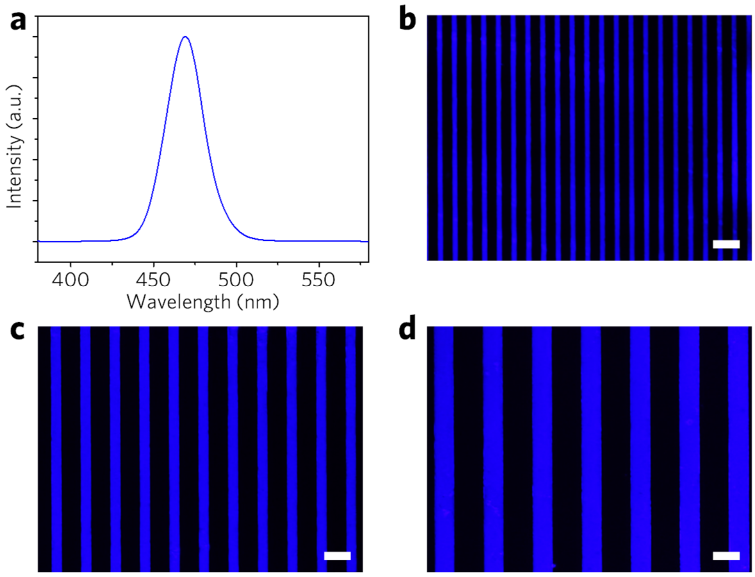

Supplementary Figure 22. Patterning of blue QDs array via SEPD. **a,** PL spectrum of blue CdZnSe/ZnS QDs terminated with PEG-COOH. **b-d,** PL images of blue QD stripe array with different linewidth and space. All scale bars, 50 μm.

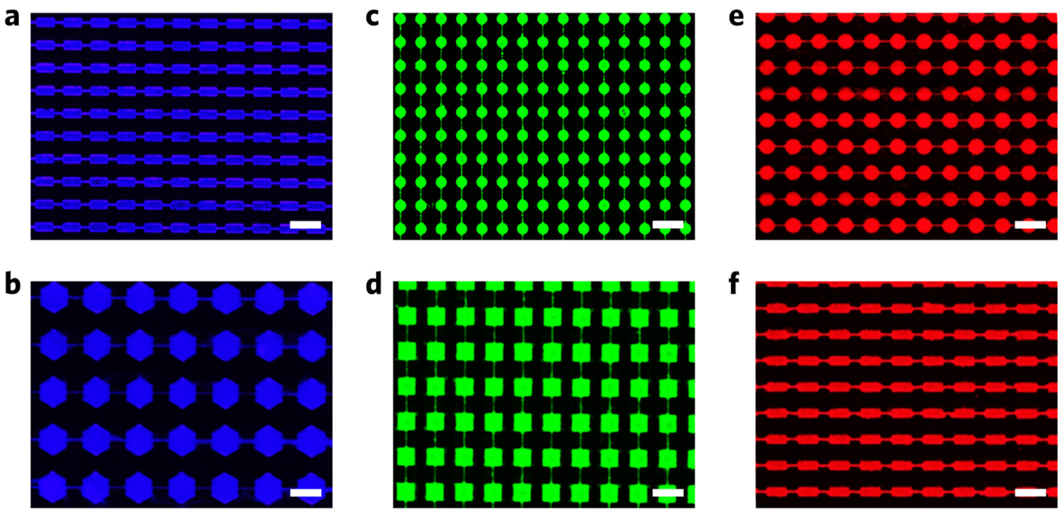

Supplementary Figure 23. Fluorescence images of array of different QD patterns under UV light. **a,** blue rectangle pattern. **b,** blue hexagon pattern. **c,** green circle pattern. **d,** green square pattern. **e,** red circle pattern. **f,** red rectangle pattern. All scale bars, 100 μm.

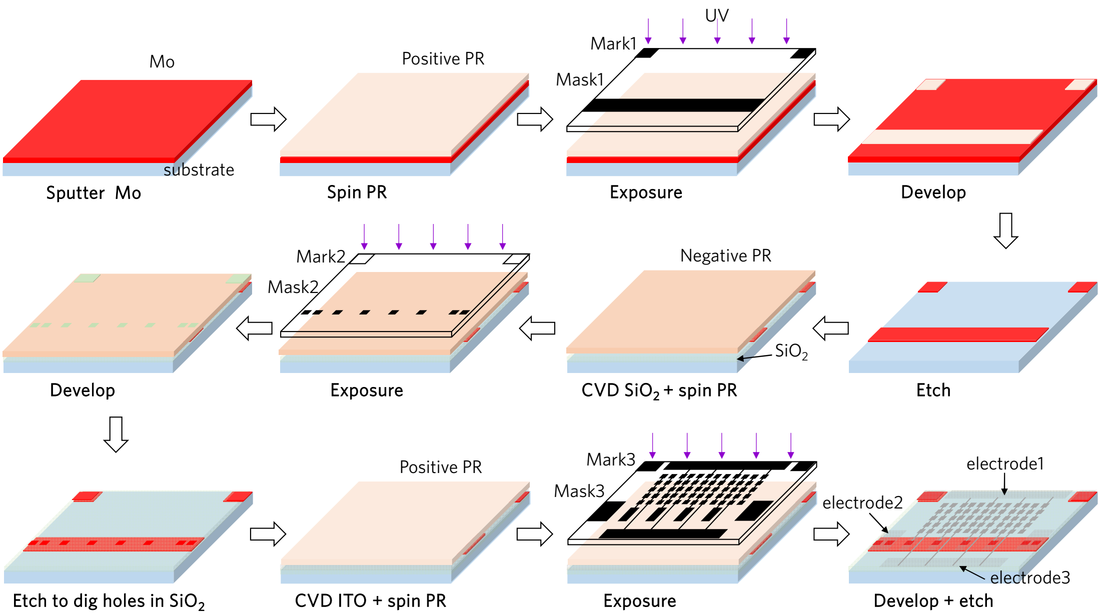

Supplementary Figure 24. Schematic illustration of preparing patterned three-electrode substrates by photolithography.

Supplementary Figure 24 shows the process of preparing patterned three-electrode substrates by photolithography. Firstly, bare glass substrates were cleaned in an ultrasonic bath using detergent and deionized water in sequence for 15 min each, and then dried in an oven at 60 °C for 30 min. Then a Mo film about 120 nm was deposited by a direct current magnetron sputtering (MSP 3200, Beijing Jinshengweina tech Co., LTD) for 690 s with a power of 100 W, an Ar flow of 50 sccm and a chamber pressure of 0.36 Pa. Then, the positive photoresist (PR) (PR1-2000A1, Futurrex, Inc.) was spun on Mo film within two steps at a speed of 800 rpm for 8 s and 3000 rpm for 30 s using a coater (KW-4B model from SETCAS Electronics Co., Ltd) and heated at 130 °C for 70 s. Then the film was exposed to UV light about 11.3 s under a mask (mask 1) using an exposure equipment (URE-2000A from Institute of Optics and Electronics, Chinese Academy of Sciences) and heated at 120 °C for 120 s. In the development and etching

process, the Mo film coated with PR was immersed in developer (DPD-200 from Dongjin Semichem Co., Ltd) for 35 s and etching liquid (150 ml 30% hydrogen peroxide solution: 300 ml water: 2 ml ammonia) for 50 s following by ultrasonic cleaning with acetone, anhydrous alcohol and deionized water for 5 min sequentially.

Subsequently, a 200 nm SiO_2 film was deposited on Mo film by chemical vapor deposition (SKE102012) with a power of 30 W, a gas flow of SiH_4:N_2O = 100 sccm: 400 sccm and a chamber pressure of 0.7 Torr. Then, the negative PR (NR9-1500PY, Futurrex, Inc.) was spun on SiO_2 film and heated at 150 °C for 70 s. Before exposure, the alignment marks of mask 2 were aligned with the Mo marks prepared in the first photolithography. Then the PR film was exposed to UV light about 26.3 s and heated at 120 °C for 65 s. In the development process, the PR on unexposed area was washed away using developer (DPD-200) for 10 s. In the dry etching process, SiO_2 film on unexposed area was removed by reactive ion etching (SKE102012, with power of 150 W, gas flow of CF_4: O_2 = 30 sccm: 5 sccm, pressure of 0.15 Torr). After that, the substrate plate was ultrasonic cleaned with acetone, anhydrous alcohol and deionized water for 5 mins sequentially. Hence, the hole array on SiO_2 film was obtained and Mo electrode was exposed from the holes.

Finally, to fabricated patterned ITO electrodes, similar with the first step, 70 nm ITO and PR (PR1-2000A1, Futurrex, Inc.) were sequentially deposited on the SiO_2 film, followed by exposure and development of the PR. Similarly, the alignment marks of mask 3 were aligned with the Mo marks. The ITO film coated with PR was immersed in developer (DPD-200) for 35 s and etching liquid (oxalic acid, 3.4%) for about 330 s

at 25 °C to get patterned ITO film. Finally, a series of electrodes were connected with the Mo electrode through the holes on the SiO_2 film. Thus three-electrode substrate was obtained. Then PR layer was removed by acetone, and the ITO film was annealed at 253 °C for 1 h to improve its conductivity.

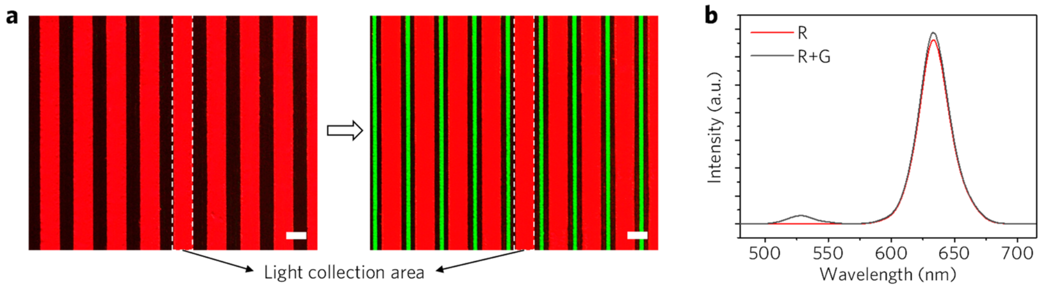

Supplementary Figure 25. Characterization of SEPD QD films affected by other QDs solution. **a, b,** PL images and spectra collected from the area marked by the dashed square shown in (**a**) of red stripe before and after depositing green QD. All scale bars, 100 μm.

The PL intensity and PLQY of SEPD QD film changed little before and after being washed with PGMEA (Supplementary Fig. 17), indicating that the luminescence properties of QD film were not affected by solvent washing. Furthermore, we used a micro-area measurement system (2D spectroradiometer, SR-5000H) to measure the PL spectra of identical red QD stripe excited by the same blue light before and after depositing green QD layers. The PL intensity of red QD stripe did not decrease with the deposition of green QDs, but slightly increased due to the absorption of green light (Supplementary Fig. 25b). All these results demonstrate the luminescence characteristics of underlying QD layers were not affected by solvent washing and subsequent deposition of another QD layer.

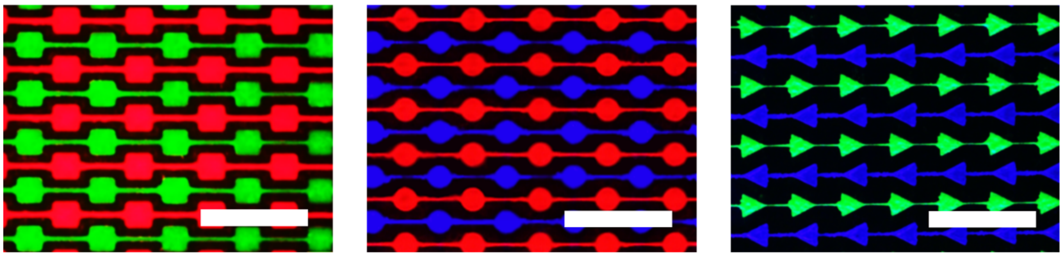

Supplementary Figure 26. Fluorescence images of multicolor QD patterns fabricated by two-step SEPD. All scale bars, 200 μm.

Supplementary Note 4. Performance of SEPD QLED

The detailed device structures are shown in **Supplementary Fig. 27**. Each functional layer possesses clear and clean boundaries, and good thickness uniformity, which would help devices to achieve high performance. Compared with some QLED via inkjet printing (IJP) (**Supplementary Fig. 28**), SEPD-QLEDs display more uniform fluorescence due to the electric field induced inhibition of coffee-ring effect and the free of capillary effect of bank structure. The SEPD QLED devices exhibit very saturated and pure colors, as demonstrated by the Commission Internationale de l'Eclairage (CIE) chromaticity diagram shown in **Supplementary Fig. 29**. The angular distribution of the SEPD-QLED shown in **Supplementary Fig. 30** indicates that the emission of QLEDs is primarily along the substrate-normal direction due to microcavity. The peak current efficiency histograms for 48 devices (**Supplementary Fig. 31**) show that the device performance is highly reproducible.

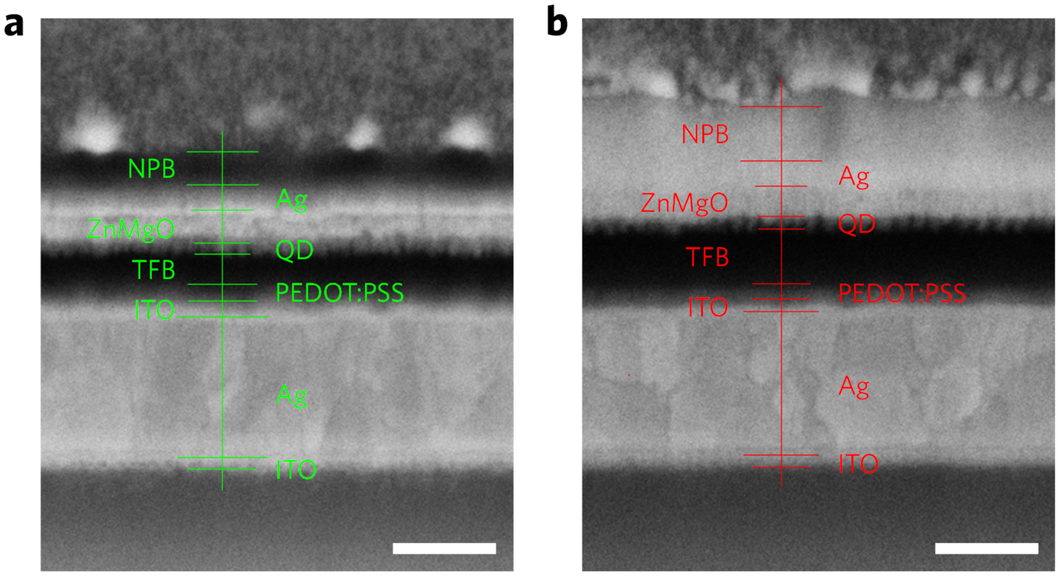

Supplementary Figure 27. Cross-sectional high-resolution STEM image of green (**a**) and red (**b**) SEPD-QLED that shows the detailed layer information of active layers. Scale bars, 100 nm.

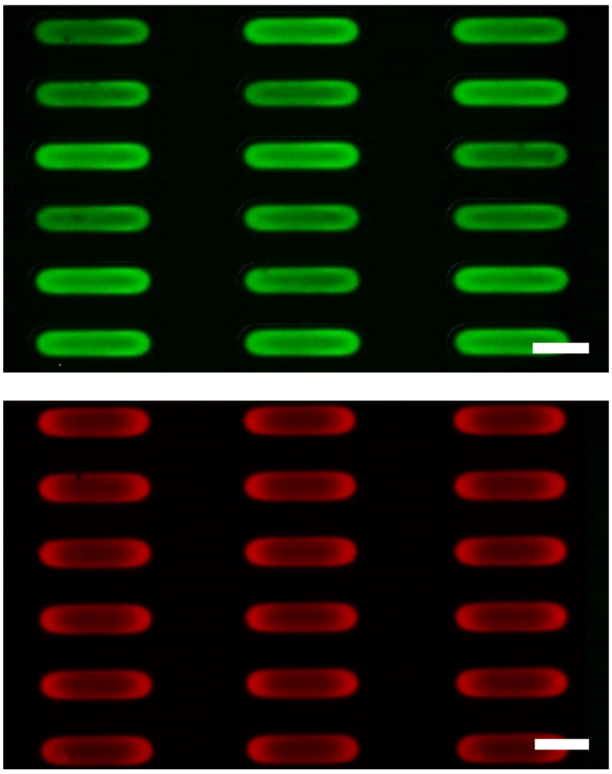

Supplementary Figure 28. EL images of green and red QLEDs pixels prepared by inkjet printing show the non-uniform electroluminescence due to the non-uniformity of QD film originated from coffee-ring effect and capillary effect of bank structure. Scale bars, 100 μm.

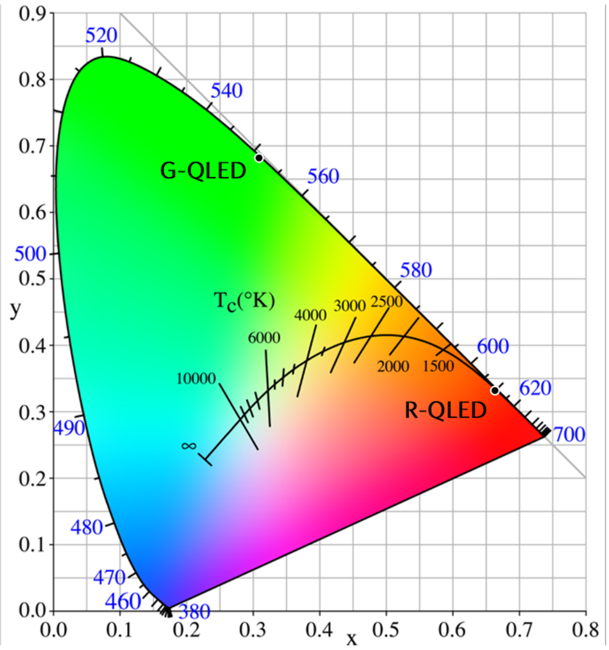

Supplementary Figure 29. CIE coordinates of green and red SEPD QLEDs.

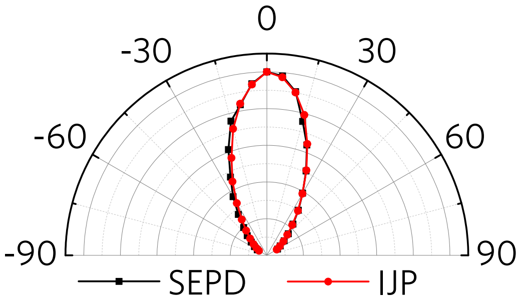

Supplementary Figure 30. The angular distribution of the radiation intensity of SEPD-QLED and IJP-QLED shows that radiation direction of QLED is mainly along the long axis of the microcavity.

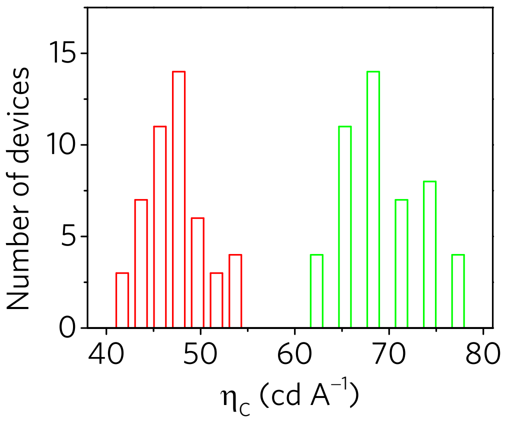

Supplementary Figure 31. Histogram of current efficiencies (η_C) of 48 devices for red and green SEPD-QLEDs. The average η_C is of 69.1 cd A^{-1} and 47.6 cd A^{-1} for green and red QLEDs, respectively.

Supplementary Note 5. Comparison of different QD patterning methods

Supplementary Table 2 shows a comparison of various patterning methods as well as our SEPD. The SEPD technique demonstrates some advantages: such as accurate control of thickness, low damage of QDs, easy implementation of both large area and high resolution, smooth morphology leading to high quality interfaces with optoelectronic devices.

Method	Resolution	Large Area Fabrication	Factors Influencing Film Uniformity	Destructiveness to QDs
**SEPD (our result)**	2 μm or less	Easy to achieve	Uniformity in the electric field	Low
**Inkjet printing**	10-50 μm[29,31]	Not efficient (need high-density nozzle arrays)[24]	Coffee ring effect[7,17]	Low
**Photolithography**	Sub-μm[31]	Easy to achieve	Uniformity of UV exposure and photoresist coating[5,6]	High[31]
**Contact transfer**	6 μm[12]	Not efficient (need many stamps)	Alignment accuracy of stamp[27], and uniformity in the stamp pressure[31]	Low
**Electrohydrodynamic jet printing**	Tens of nm[19-21]	Not efficient (need high-density nozzle arrays)[24]	Residual charges on the printed droplets[24]	Low

Supplementary Table 2. Comparison of different QD patterning methods

Research efforts over the last several years have led to significant progresses on patterning quantum dots with technologies including photolithography[3-7], contact printing[8-13], inkjet printing[14-18], and electrohydrodynamic printing[19-25]. High-resolution, large-area and full color patterning has been demonstrated[26-28].

Previous photolithography-related approaches have partially resolved the scaling and uniformity issues for large area patterning, but there are still two main difficulties for application of QDs patterning with photolithography. First, general photolithographic technology limits QD loading due to low dispersibility of QDs in common photoresist[4,5,29]. Second, most of the chemicals used in the photolithography processes are organic solvents that could damage or dissolve precoated QD layers because most of conventional colloidal QDs are functionalized with hydrophobic or nonpolar ligands to achieve high internal quantum efficiency[5,6,26]. Wang and co-workers

promoted direct optical lithography of functional inorganic nanomaterials (DOLFIN) to achieve patterning with light-responsive ligands[30]. However, the ligand exchange process with DOLFIN induced significant drops in the photoluminescence quantum yield (PLQY)[31]. In other reports, researchers combined conventional photolithography with a layer-by-layer (LBL) assembly process to micropattern of colloidal QDs[26,32]. Though the damage to preceding QD patterns is alleviated, the repeating deposition process of LBL method is lack of efficiency.

Contact printing method has been developed with a demonstration of high-resolution, multicolored QD patterns on a single substrate. The process can be conducted in ambient condition, and is solvent free[13]. However, the material loss is still high with a pre-prepared film[18]. Both the resolution and area of patterned QDs are limited by the size and pressure uniformity of the elastomer stamp[26,29]. It also becomes difficult to pattern QD clusters on a pre-defined structure with relatively good alignment[9-11]. Therefore, process complexity and alignment accuracy of repetitive transfer are challenging for contact printing method.

Inkjet printing (IJP) and electrohydrodynamic printing (EHD) are both promising candidates for QD patterning due to mild patterning conditions, mask-free process, material saving and noncontact printing[14]. The key target of IJP is to reduce the common inhomogeneity such as the coffee-ring effect. Successful jetting without coffee ring is highly dependent on the viscosity and vapor pressure of the solvent, whose choice is thus much limited[29]. The ultimate surface morphology of patterns from jetting are

affected by formulation of ink, nozzle status and post treatment requiring complex process control.

EHD utilizes a jet with a much smaller orifice (about 1-5 μm) and the DC bias between the nozzle and the substrate generates droplets 10 times smaller than the opening. Much smaller droplets alleviate nozzle clogging in printing viscous or high-density particle suspensions[24,29]. This method offers better performance in terms of resolution, registration, and thickness control that are vastly superior to IJP[20]. But it needs a high electric field between substrate and nozzle to eject ink from nozzle, causing issues in stability and repeatability[17]. A build-up of residual charges on the printed droplets can alter the dynamics of printing, particularly on insulating substrates. So electrostatic interference between the nozzles must be considered in EHD[24]. Furthermore, truly high-throughput printing for thick film and large area requires the development of large nozzle array print-head fabrication for both IJP and EHD.

As discussed above, challenges remain in achieving large area, high-PPI and semiconductor-manufacturing-compatible fabrication of patterning QDs. By integrating both photolithography and EPD technology, SEPD possesses high precision of photolithography and high throughput of solution processing, which suggests a manufacture-viable technology to construct large area and high-PPI QDs patterning. The SEPD method favors the following advantages: accurate control of thickness, low damage of QDs, easy implementation of both large area and high resolution, and smooth morphology (depending the electric field uniformity) benefiting high quality interfaces in constructing optoelectronic devices. Moreover, it is compatible to

semiconductor photolithographic processes suitable for mass fabrication. Supplementary Table 2 shows the comparison of various patterning methods discussed here.

Supplementary References

1. Chen, K. J. *et al.* White light emitting diodes with enhanced CCT uniformity and luminous flux using ZrO_2 nanoparticles. *Nanoscale* **6**, 5378-5383 (2014).
2. Sugimoto, H., Ozaki, Y. & Fujii, M. Silicon quantum dots in dielectric scattering media: broadband enhancement of effective absorption cross section by light trapping. *ACS Appl. Mater. Interfaces* **9**, 19135–19142 (2017).
3. Rodríguez-Cantó, P. J. *et al.* UV-patternable nanocomposite containing CdSe and PbS quantum dots as miniaturized luminescent chemo-sensors. *RSC Adv.* **5**, 19874. (2015).
4. Menon, V. M., Husaini, S., Okoye N. & Vallapil, N. V. Integrated photonics using colloidal quantum dots. *Nanophotonics 3,* 031608 (2009).
5. Mei, W. *et al.* High-resolution, full-color quantum dot light-emitting diode display fabricated via photolithography approach. *Nano Res.* **13**, 2485-2491 (2020).
6. Park, J. J. *et al.* Photopatternable quantum dots forming quasi-ordered arrays. *Nano Lett.* **10**, 2310-2317 (2010).
7. Ko, J. *et al.* Direct photolithographic patterning of colloidal quantum dots enabled by uv-crosslinkable and hole-transporting polymer ligands. *ACS Appl. Mater. Interfaces* **12***,* 42153-42160 (2020).
8. Kim, L. *et al.* Contact printing of quantum dot light-emitting devices. *Nano Lett.* **8**, 4513-4517 (2008).
9. Park, Y. *et al.* Nanoscale patterning of colloidal quantum dots on transparent and metallic planar surfaces. *Nanotechnology* **23**, 355302 (2012).
10. Yan, X. *et al.* Microcontact printing of colloidal crystals. *J. Am. Chem. Soc.* **126**, 10510-10511 (2004).
11. Kim, T.-H. *et al.* Full-colour quantum dot displays fabricated by transfer printing. *Nat. Photon.* **5**,

176-182 (2011).

12. Choi, M. K. *et al.* Wearable red-green-blue quantum dot light-emitting diode array using high-resolution intaglio transfer printing. *Nat. Commun.* **6**, 7149 (2015).

13. Qin, D., Xia, Y. & Whitesides, G. M. Soft lithography for micro- and nanoscale patterning. *Nat. Protoc.* **5**, 491-502 (2010).

14. Lan, L. *et al.* Inkjet printing for electroluminescent devices: emissive materials, film formation, and display prototypes. *Front. Optoelectron.* **10**, 329–352 (2017).

15. Tekin, E., Smith, P. J. & Schubert, U. S. Inkjet printing as a deposition and patterning tool for polymers and inorganic particles. *Soft Matter.* **4**, 703-713 (2008).

16. Singh, M. *et al.* Inkjet printing-process and its applications. *Adv. Mater.* **22**, 673-685 (2010).

17. Jiang, C. *et al.* Coffee-ring-free quantum dot thin film using inkjet printing from a mixed-solvent system on modified zno transport layer for light-emitting devices. *ACS Appl. Mater. Interfaces* **8**, 26162-26168 (2016).

18. Haverines, H. M., Myllyla, R. A. & Jabbour, G. E. Inkjet printed RGB quantum dot-hybrid LED. *J. Disp. Technol.* **6**, 87-89 (2010).

19. Richner, P. *et al.* Full-spectrum flexible color printing at the diffraction limit. *ACS Photon.* **3**, 754-757 (2016).

20. Kim, B. H. *et al.* High-resolution patterns of quantum dots formed by electrohydrodynamic jet printing for light-emitting diodes. *Nano Lett.* **15**, 969-973 (2015).

21. Kress, S. J. P. *et al.* Near-field light design with colloidal quantum dots for photonics and plasmonics. *Nano Lett.* **14**, 5827-5833 (2014).

22. Richner, P., Kress, S. J. P., Norris, D. J. & Poulikakos, D. Charge effects and nanoparticle pattern

formation in electrohydrodynamic nanodrip printing of colloids. *Nanoscale* **8**, 6028-6034 (2016).

23. Galliker, P., Schneider, J. & Poulikakos, D. Dielectrophoretic bending of directly printed free-standing ultra-soft nanowires. *Appl. Phys. Lett.* **104**, 073105 (2014).

24. Onses, M. S., Sutanto, E. & Ferreira, P. M. Mechanisms, capabilities, and applications of high-resolution electrohydrodynamic jet printing. *Small* **11**, 4237-4266 (2015).

25. Galliker, P. *et al.* Direct printing of nanostructures by electrostatic autofocussing of ink nanodroplets. *Nat. Commun.* **3**, 890 (2012).

26. Park, J. S. *et al.* Alternative patterning process for realization of large-area, full-color, active quantum dot display. *Nano Lett.* **16**, 6946-6953 (2016).

27. Kang, H. L. *et al.* Spatial light patterning of full color quantum dot displays enabled by locally controlled surface tailoring. *Adv. Opt. Mater.* **6**, 1701335 (2018).

28. Nam, T. W. *et al.* Thermodynamic-driven polychromatic quantum dot patterning for light-emitting diodes beyond eye-limiting resolution. *Nat. Commun.* **11**, 3040 (2020).

29. Smith, M. J. *et al.* Composite structures with emissive quantum dots for light enhancement. *Adv. Opt. Mater.* **7**, 1801072 (2019).

30. Wang, Y. Y., Fedin, I., Zhang, H. & Talapin, D. V. Direct optical lithography of functional inorganic nanomaterials. *Science* **357**, 385-388 (2017).

31. Cho, H. *et al.* Direct optical patterning of quantum dot light-emitting diodes via in situ ligand exchange. *Adv. Mater.* **32**, 2003805 (2020).

32. Lin, C. H. *et al.* Large-scale robust quantum dot microdisk lasers with controlled high quality cavity modes. *Adv. Opt. Mater.* **5**, 1700011 (2017).
