# Supplementary material for: Large-area patterning of full-color quantum dot arrays beyond 1000 pixels per inch by selective electrophoretic deposition
Source: Nat Commun. 2021 Jul 29;12:4603. doi: 10.1038/s41467-021-24931-x (PMC8322170; doi:10.1038/s41467-021-24931-x)
Supplement: Supplementary file 2 — Description of Additional Supplementary Files [file 41467_2021_24931_MOESM2_ESM.pdf]

Description of Additional Supplementary Files

File Name: Supplementary Movie 1
Description: The movie of SEPD of QDs in solution.
